# Author Correction: Effect of gut microbiome modulation on muscle function and cognition: the PROMOTe randomised controlled trial

**DOI:** 10.1038/s41467-025-58771-w

**Published:** 2025-04-10

**Authors:** Mary Ni Lochlainn, Ruth C. E. Bowyer, Janne Marie Moll, María Paz García, Samuel Wadge, Andrei-Florin Baleanu, Ayrun Nessa, Alyce Sheedy, Gulsah Akdag, Deborah Hart, Giulia Raffaele, Paul T. Seed, Caroline Murphy, Stephen D. R. Harridge, Ailsa A. Welch, Carolyn Greig, Kevin Whelan, Claire J. Steves

**Affiliations:** 1 King’s College London, Department of Twin Research and Genetic Epidemiology, London, SE1 7EH UK; 2 The Alan Turing Institute, London, NW1 2DB UK; 3 Clinical Microbiomics, Copenhagen, Denmark; 4 GKT School of Medical Education, King’s College London, London, UK; 5 Unit for Medical Statistics/Department for Women and Children’s Health, School of Life Course and Population Sciences, Faculty of Life Sciences and Medicine, King’s College London, London, UK; 6 King’s Clinical Trials Unit, Research Management and Innovation Directorate, King’s College London, London, UK; 7 Centre for Human & Applied Physiological Sciences, King’s College London, London, UK; 8 Department of Epidemiology and Public Health, Norwich Medical School, University of East Anglia, Norwich, NR4 7TJ UK; 9 School of Sport, Exercise, and Rehabilitation Sciences, University of Birmingham, Birmingham, UK; 10 MRC-Versus Arthritis Centre for Musculoskeletal Ageing Research, Birmingham, UK; 11 NIHR Birmingham Biomedical Research Centre, University Hospitals Birmingham NHS Foundation Trust and University of Birmingham, Birmingham, UK; 12 King’s College London, Department of Nutritional Sciences, Franklin Wilkins Building, SE1 9NH London, UK

**Keywords:** Risk factors, Translational research

Correction to: *Nature Communications* 10.1038/s41467-024-46116-y, published online 29 February 2024

In the version of the article initially published, in the “Microbiota features and muscle strength” section, the text “…144 microbiota features were significantly correlated with chair rise time, including 109 microbial taxa, 33 microbial functions” has been corrected to “… 129 microbiota features were significantly correlated with chair rise time, including 95 microbial taxa, 32 microbial functions”. In the “Microbiota features and cognition” section, “eight” has been corrected to “five” in the text now reading “…we observed five microbiota features that correlated significantly with cognitive ability”. In the “Gut microbiome” section, the sentence “Sixty microbiome features changed between baseline and study end for the prebiotic group, while only three were changed in placebo” has been corrected to “Forty microbiome features changed between baseline and study end for the prebiotic group, while only one was changed in placebo”. Supplementary Figs. 6, 8 and 10 have also been amended accordingly. In the “Study Interventions” section, the text “The intervention arm sachets also contained 7.5 g of prebiotic (Darmocare Pre®, Bonsuvan), which consists of inulin (3.375 mg) and fructo-oligosaccharides (FOS) (3.488 mg)” should have read “The intervention arm sachets also contained 7.5 g of prebiotic (Darmocare Pre®, Bonusan), which consists of inulin (min. 3375 mg) and fructo-oligosaccharides (FOS) (min. 3488 mg).”

