# Supplementary material for: Effect of gut microbiome modulation on muscle function and cognition: the PROMOTe randomised controlled trial
Source: Nat Commun. 2024 Feb 29;15:1859. doi: 10.1038/s41467-024-46116-y (PMC10904794; doi:10.1038/s41467-024-46116-y)
Supplement: Supplementary file 1 — Supplementary Information [file 41467_2024_46116_MOESM1_ESM.pdf]

Effect of gut microbiome modulation on muscle function and cognition: the PROMOTe randomised controlled trial

Supplementary Information

Contents:

1. Supplementary Notes
2. Supplementary Figures
3. Supplementary Tables
4. References
5. PROMOTe Study protocol
6. PROMOTe Study Statistical analysis plan

1 Supplementary Note

1.1 *Supplementary Note 1: Detailed Methodology and Results of Gut Microbiota Measures*

1.1.1 Methods

1.1.1.1 DNA sequencing

Before sequencing, the quality of the DNA samples was evaluated using agarose gel electrophoresis and the quantity of the DNA was evaluated by Qubit 2.0 fluorometer quantitation. The genomic DNA was randomly sheared into fragments of around 350 bp. The fragmented DNA was used for library construction using NEBNext Ultra II Library Prep Kit for Illumina (New England Biolabs). The prepared DNA libraries were evaluated using Qubit 2.0 fluorometer quantitation and Agilent 2100 Bioanalyzer for the fragment size distribution. Quantitative real-time PCR (qPCR) was used to determine the concentration of the final library before sequencing. The library was sequenced using 2 x 150 bp paired-end sequencing on an Illumina platform.

1.1.1.2 Gene catalogue and MGS definitions

We used a reference gene catalogue with 14 355 839 genes created from 21 598 human gut specimens (including 481 from infants, 9 428 publicly available metagenomes compiled from 43 countries[1],) and 3 567 publicly available genome assemblies) from isolated microbial strains. For taxonomic abundance profiling, we used Clinical Microbiomics HGMGS version HG4.D.2 metagenomic species (MGS), each represented by a set of genes with highly coherent abundance profiles and base compositions. The metagenomic species concept is described in [2].

To taxonomically annotate an MGS, we blasted its genes against NCBI RefSeq prokaryotic genomes (2022-01-19) and nt (2021-08-03) databases and used rank-specific annotation criteria. Specifically, we assigned a taxon to an MGS if at least M % of its genes were mapped to the taxon and no more than D % of its genes were mapped to a different taxon. We only considered blast hits with an alignment length ≥ 100 bp, ≥ 50 % query coverage and % identity ≥ PID. Here we define: PID = (95, 95, 85, 75, 65, 55, 50, 45); M = (75, 75, 60, 50, 40, 30, 25, 20); and D = (10, 10, 10, 20, 20, 20, 20, 15) for subspecies, species, genus, family, order, class, phylum, and superkingdom, respectively. Finally, we processed each MGS with CheckM [3], and updated our annotation with the CheckM result if this resulted in a lower taxonomic rank.

1.1.1.3 Sequencing data pre-processing

Raw FASTQ files were filtered to remove host contamination by discarding read pairs in which either read mapped to the human reference genome GRCh38 with Bowtie2 (v. 2.4.2, [4]). Reads were then trimmed to remove adapters and

bases with a Phred score below 20 using AdapterRemoval (v. 2.3.1, [5]). Read pairs in which both reads passed filtering with a length of at least 100 bp were retained; these were classified as high-quality non-host (HQNH) reads.

1.1.1.4 Mapping reads to the gene catalogue

HQNH reads were mapped to the gene catalogue using BWA mem (v. 0.7.17) ([6]). An individual read was considered uniquely mapped to a gene if the mapping quality (MAPQ) was ≥ 20 and the read aligned with ≥ 95 % identity over ≥ 100 bp. However, if > 10 bases of the read did not align to the gene or extend beyond the gene, the read was considered unmapped. Reads meeting the alignment length and identity criteria but not the MAPQ threshold were considered multi-mapped.

Each read pair was counted as either 1) uniquely mapped to a specific gene, if one or both individual reads were uniquely mapped to a gene, or 2) multi-mapped, if neither read was uniquely mapped, and at least one was multi-mapped, or 3) unmapped, if both individual reads were unmapped. If the two reads were each uniquely mapped to a different gene, the gene mapped by read 1 was counted but not the gene mapped by read 2. A gene count table was created with the number of uniquely mapped read pairs for each gene.

1.1.1.5 MGS relative abundance calculation

For each MGS, a signature gene set was defined as the 100 genes optimized for accurate abundance profiling of the MGS. An MGS count table was created by counting the number of reads uniquely mapped to the MGS signature genes per sample. An MGS was considered detected if reads from a sample uniquely mapped to at least three of its signature genes; measurements that did not satisfy this criterion were set to zero. Based on internal benchmarks, this threshold results in 99.6 % specificity. The MGS count table was normalized according to effective gene length and then normalized sample-wise to sum to 100 %, resulting in relative abundance estimates for each MGS.

Down sampled (rarefied) MGS abundance profiles were calculated by random sampling, without replacement, of a fixed number of signature gene counts per sample, and then following the procedure described above. In this study, 431127 signature gene counts were sampled.

1.1.1.6 Functional annotation and profiling

EggNOG-mapper (v. 2.0.1, Diamond mode), [7] was used to map each gene in the gene catalogue to the EggNOG (v. 5.0) orthologous groups database, resulting in EggNOG annotations for 79 % of genes and Kyoto Encyclopaedia of Genes and Genomes (KEGG) orthology (KO) database annotations for 46 % of genes. Functional potential profiles based on KOs were calculated as the proportion of all mapped reads that mapped to a given KO. KEGG modules (v. 78.2) [8] are defined as a set of Kos that enable a specific function or pathway. For each KEGG module, we define its corresponding FSG as the set of MGSs that include at least 2/3 of the genes that encode the proteins/enzymes that are needed to complete the functionality of the module. If a module has alternative reaction paths, only one of

these is required to be 2/3 complete. For modules with three or fewer steps, all steps are required to be comprised in the MGS.

The Gut Metabolic Modules (GMMs) are a set of 103 conserved metabolic pathways, each defined as a series of enzymatic steps represented by KO identifiers [9]. We consider an MGS to contain a given module if the MGS includes genes annotated to at least 2/3 of the KOs needed to complete the functionality of the module. If a module has alternative reaction paths, only one of these is required to be 2/3 complete. For modules with three or fewer steps, all steps are required to be comprised in the MGS.

The Gut Brain Modules (GBMs) are a set of 56 microbial pathways for metabolizing neuroactive compounds (molecules that have the potential to interact with the human nervous system). Each GBM corresponds to a single neuroactive compound synthesis or degradation process by members of the gut microbiota and is defined as a series of enzymatic steps represented by orthologue group identifiers (KEGG, TIGRFAM, and eggNOG version 3.0 orthology databases in order of preference) [10]. We consider an MGS to contain a given module if the MGS includes genes annotated to at least 2/3 of the orthologous needed to complete the functionality of the module. If a module has alternative reaction paths, only one of these is required to be 2/3 complete. For modules with three or fewer steps, all steps are required to be comprised in the MGS. Since the GBMs were defined using eggNOG v 3.0, the seed orthologs for non-supervised orthologous groups were identified in eggNOG v 3.0 (http://eggnog.embl.de/version_3.0/) and used to transfer these orthologs groups to the genes, within our gene catalogue, annotated with eggNOG v 5.0.

1.1.1.7 Diversity estimates

Alpha and beta diversity estimates were calculated from rarefied abundance matrices, created by random sampling of reads without replacement. Within each data type (e.g., gene, MGS), all samples were represented by the same number of informative sequencing reads: rarefaction of MGS abundance was performed by sampling only from reads mapping to MGS signature genes, and rarefaction of Kyoto Encyclopaedia of Genes and Genomes orthology (KO) abundance was performed by sampling only from reads mapped to a gene with an assigned KO. However, rarefaction of gene abundance was performed by sampling reads mapped to the entire gene catalogue. Alpha diversity was calculated as the number of entities detected (richness), as the Shannon index based on natural logarithm, or as Faith’s phylogenetic diversity. Beta diversity was calculated as the Bray–Curtis dissimilarity and weighted UniFrac distances.

1.1.1.8 Statistical Analysis

Testing for differences in microbiome taxon abundances was performed with a linear regression framework with a compositional bias correction based on LinDA [11]. For each sample, the relative taxon abundances were transformed with a centered log-ratio transformation to account for the compositional structure of the data. To avoid dividing by zero in the transformation, and due to the sparsity of the data, and because we omit samples missing a given taxon

from differential abundance analysis, abundances of zero were replaced with a pseudo-count equivalent to half of the lowest non-zero relative abundance observed for the taxon. For each taxon, a linear regression model was fitted with the taxon as the outcome. Any bias in the coefficients arising from the compositional structure of the taxonomical abundance data was identified and adjusted for based on the assumption that the modal regression coefficient across all taxa should be zero [11]. Bias correction was not used for functional data (GMMs and GBMs) and alpha-diversity metrics as these data are not directly compositional as is the case of the taxonomical abundance data.

The reported effect sizes are the bias-corrected coefficients from the linear models. Due to the log transformation of the data, the coefficients represent log2 fold changes for group comparison. For correlation to physical and cognitive ability, effect sizes were used to calculate (partial) Pearson correlations. Pairwise comparisons between study groups were performed by contrasting the estimated marginal means for each study group with the R package "emmeans". To be robust against outliers in the data, winsorization was performed for taxonomical and functional abundance data, capping the abundances at their 97th percentile. Taxa present in less than 10% of the samples in each pairwise comparison were not tested.

Testing for differences in microbiome taxa prevalence, i.e., whether the taxon is present or absent, was performed with a binomial generalized linear regression model. To account for differences in sequencing depth between samples, the sequencing depth per sample was included as a covariate in the model.

Pairwise comparisons of microbiota (dis)similarity were compared using the Mann-Whitney U test and verified by comparing the Mann-Whitney U test statistic obtained when analysing data with the original group labels to the test statistic distribution obtained by 1,000 group label permutations. The latter comparison was also performed using the Mann-Whitney U test.

Unless otherwise stated, permutational multivariate analysis of variance (PERMANOVA) tests were performed using the adonis2 function from the vegan R package with 1000 permutations and by = “margin”, thus assessing the marginal effects of the terms (i.e. each marginal term analysed in a model with all other variables). Multivariate homogeneity of group dispersions was tested using the betadisper and permutest functions from the vegan R package with 1000 permutations and type = “spatial median”, which is an implementation of the PERMDISP2 procedure from [12]. Distance-based redundancy analysis (dbRDA) tests were performed using the capscale function from the vegan R package with 1000 permutations and “sqrt = T” to prevent negative eigenvalues. With 1000 permutations, the lowest possible P value is 1/1001 = 0.000999.

When performing statistical testing on multiple hypotheses, we used the Benjamini–Hochberg (BH) method to control the false discovery rate (FDR) at a level of 10%. Thus, of all the “statistically significant” associations we report in this context, we expect 10 % of these to be false associations (arising due to chance). When we report the “FDR-adjusted P value”, this is not a P value per se, but rather indicates the FDR at which a given hypothesis would be called significant using the BH method. FDR control was applied to each tested contrast individually, i.e., it accounts for multiple species being tested but does not account for multiple contrasts and across multiple analysed

visits. When we refer to a “nominal” P value, this indicates that the P value is not subjected to FDR control nor otherwise adjusted for multiple hypotheses.

Broad-sense heritability was determined with ACE model (from R package mets). The model estimates how much the phenotype variation stems from additive genetic effects (A), common environment (C) and unique environment for each twin (E). The model was fitted separately to the abundances of taxa found in more than 2 samples in each arm ($abundance \sim 1$) and used to extract broad-sense heritability.

1.2 Supplementary Note 2: Factor Analysis of CANTAB Cognitive Tests

1.2.1.1 Factor analysis/correlation, Method: principal factors

Factor	Eigenvalue	Difference	Proportion	Cumulative
Factor1	2.39168	2.10609	0.9194	0.9194
Factor2	0.28559	0.05551	0.1098	1.0292
Factor3	0.23008	0.23512	0.0884	1.1177
Factor4	-0.00504	0.07484	-0.0019	1.1157
Factor5	-0.07988	0.14125	-0.0307	1.085
Factor6	-0.22113	.	-0.085	1

1.2.1.2 Factor loadings (pattern matrix) and unique variances:

Variable	Factor1	Uniqueness
Executive function (OTS)	0.213	0.9546
PAL: first attempt memory score	0.9439	0.1090
PAL: total errors	0.9183	0.1568
Pattern recognition memory	0.5116	0.7382
Spatial span	0.4875	0.7624
Spatial working memory	0.3357	0.8873

1.2.1.3 Scoring coefficients

Variable	Factor1 Scoring coefficients (method = regression)
Executive function (OTS)	0.02319
PAL: first attempt memory score	0.5411
PAL: total errors	0.35486
Pattern recognition memory	0.05789
Spatial span	0.10573
Spatial working memory	0.00626

CANTAB: Cambridge Neuropsychological Test Automated Battery. OTS: One Touch Stockings of Cambridge cognitive test. PAL: Paired Associates Learning.

2 Supplementary Figures

2.1 Supplementary Figure S1: Read Quality and Read Mapping for all Microbiome Samples.

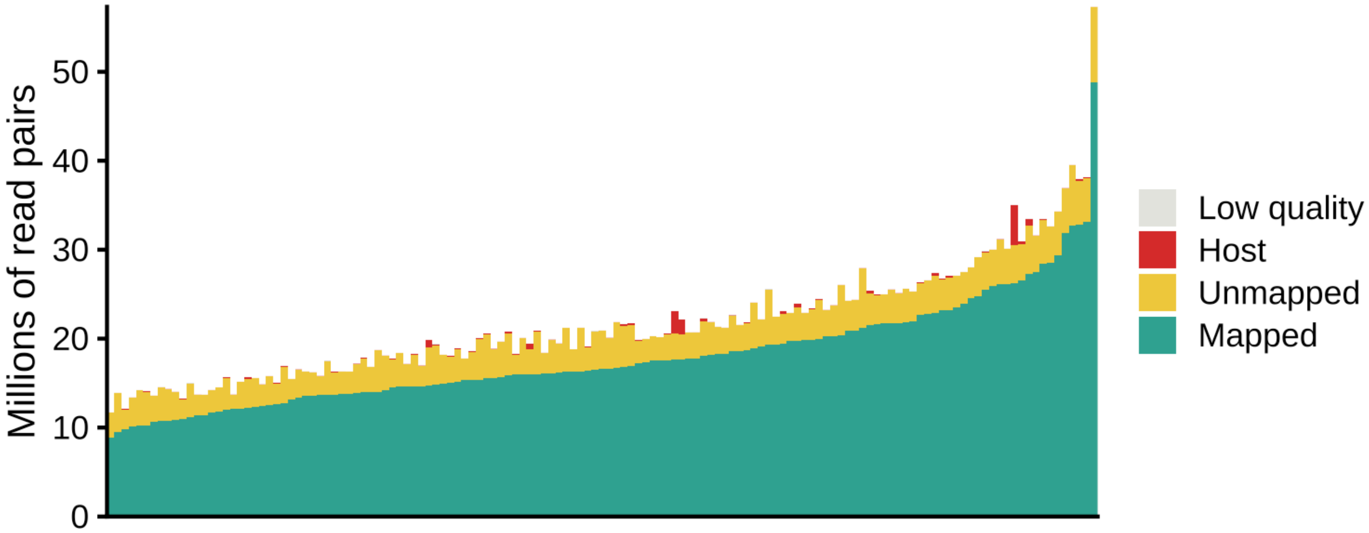

Barplots summarizing read quality and read mapping for all samples. M = millions of reads. Samples are ordered by increasing number of mapped reads.

2.2 *Supplementary Figure S2: Broad Sense Heritability of Taxa*

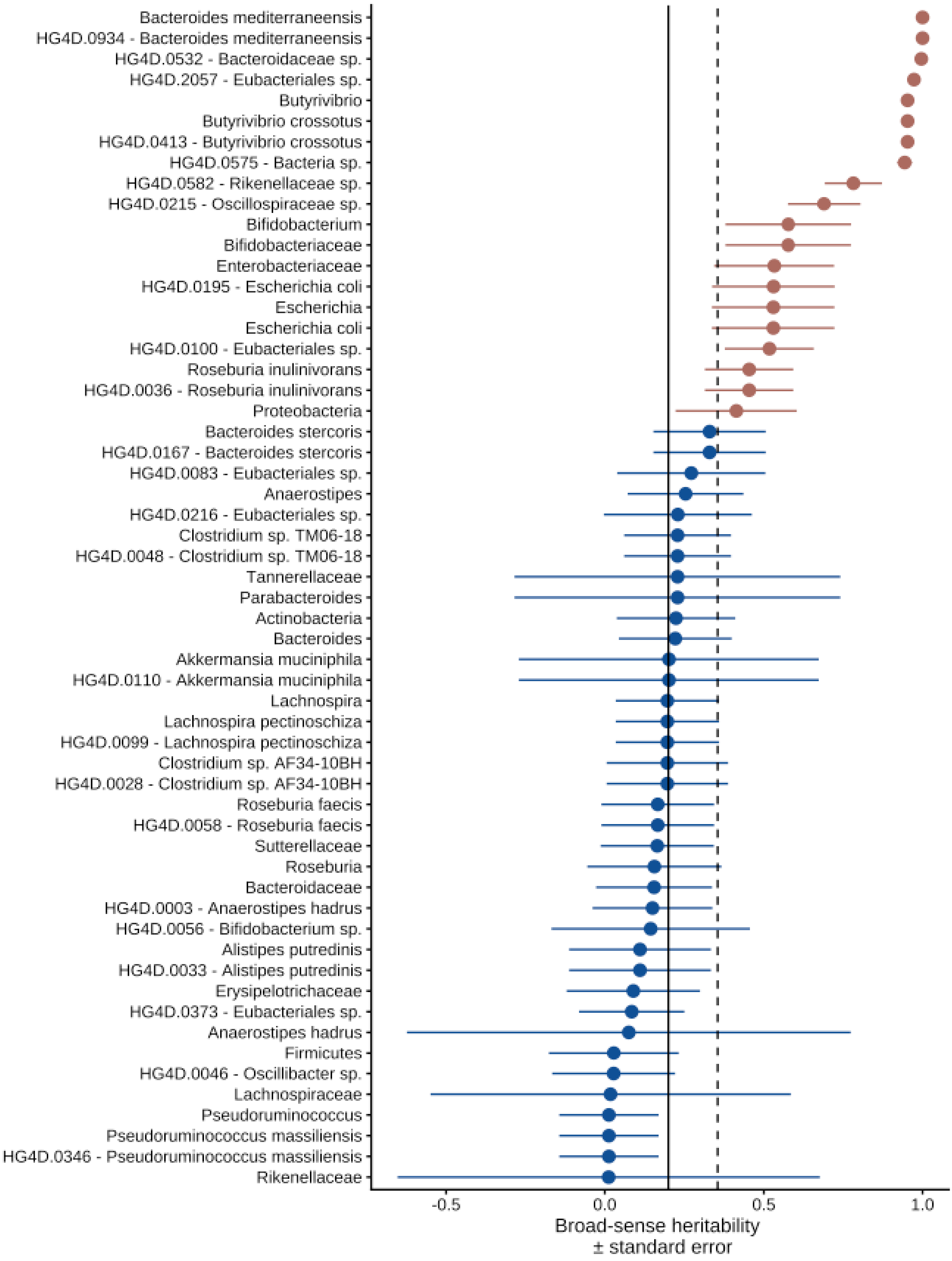

The model was fitted to the relative abundance of different taxa ($abundance \sim 1$). Only taxa with broad-sense heritability larger than 0.01 and with prevalence of 2 in twin-pairs were chosen to be included in the figure. The solid line marks the heritability value of 0.2, and the dashed line marks the mean heritability of the taxa shown in the figure. Taxa with heritability value > 0.2 are shown in brown.

2.3 Supplementary Figure S3: Taxonomic overview at genus level per sample facetted by twin pair, and by study arm.

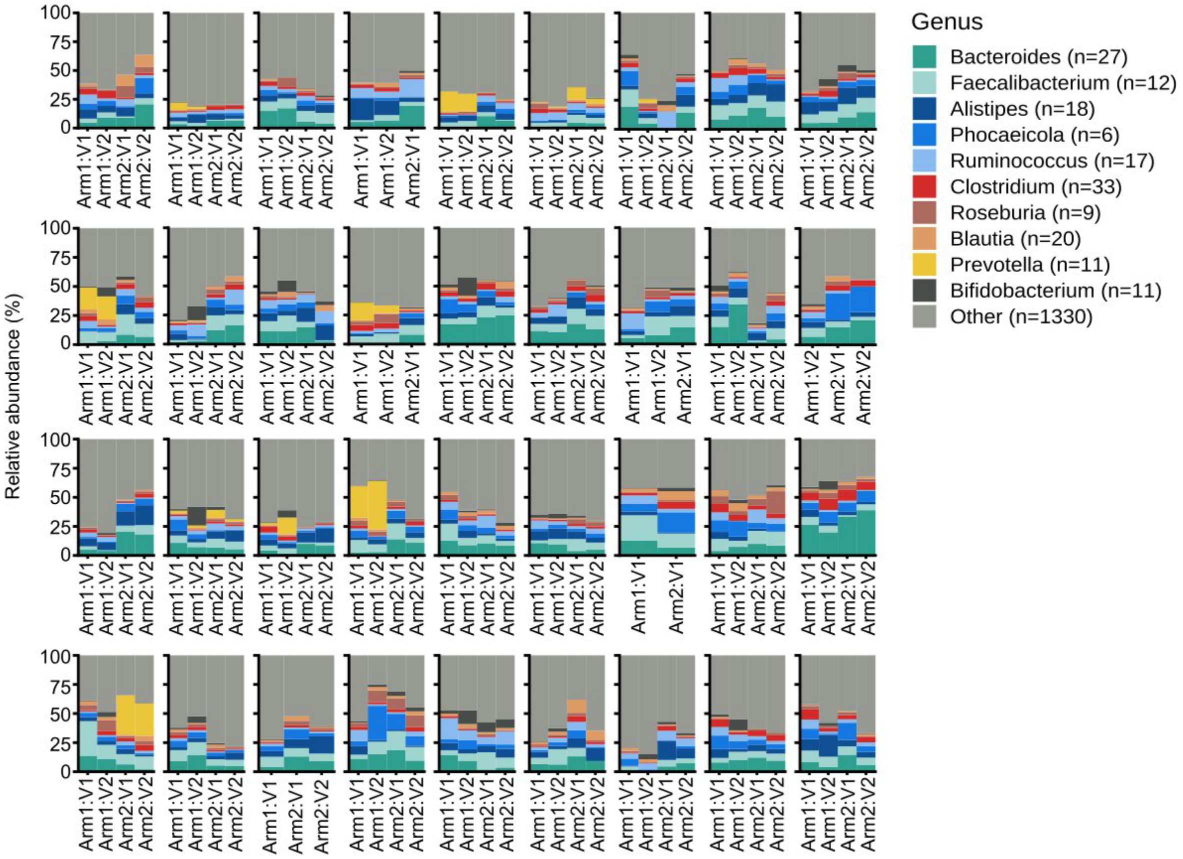

Bar plots display the relative abundance of the top 10 taxa with highest average abundance across all samples. Light grey (Other) indicates the total relative abundance of metagenomic species that are not in the top 10 most abundant taxa.

2.4 Supplementary Figure S4: Bray–Curtis dissimilarities among samples stratified based upon whether they are from the same twin pair and from disparate twins pairs

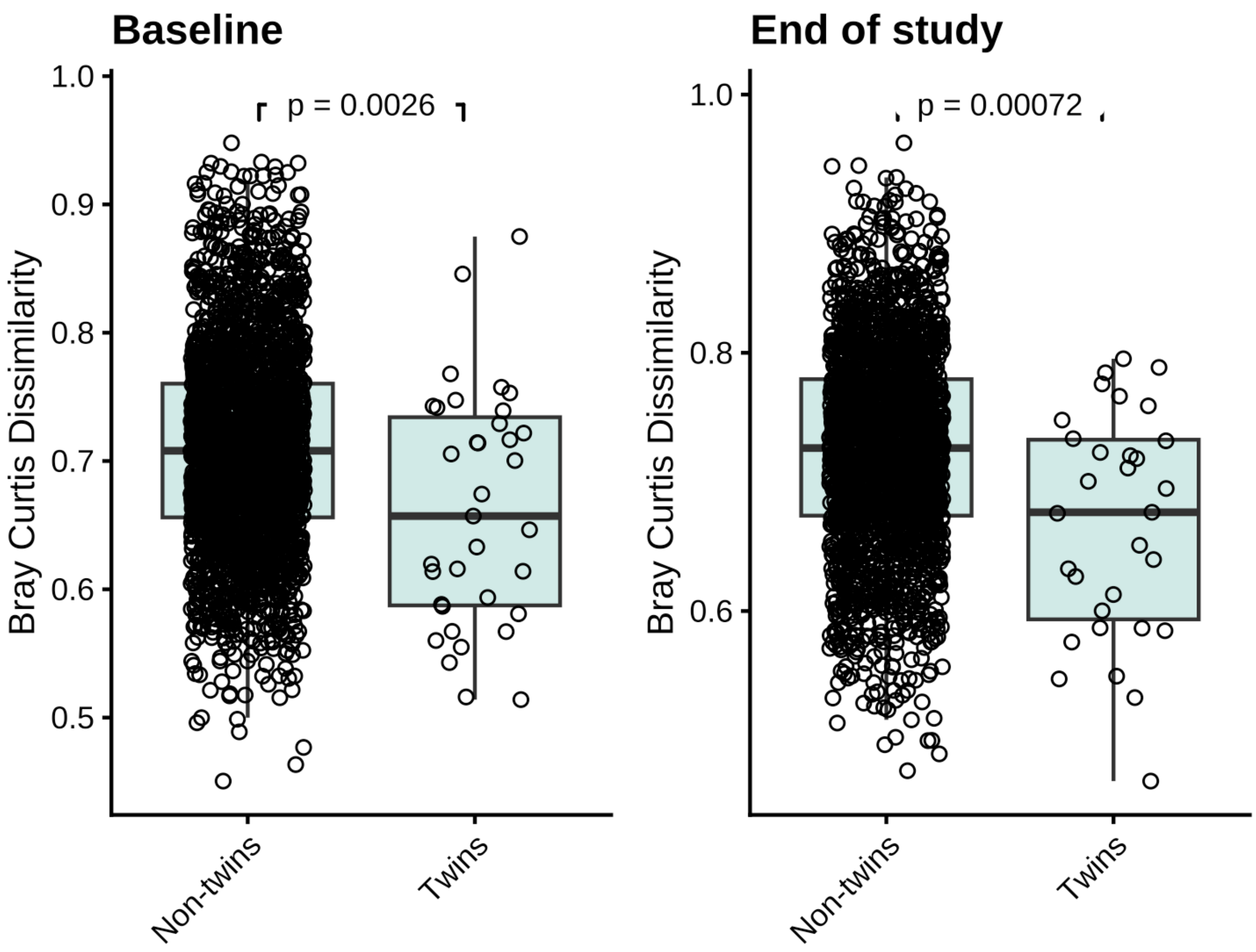

Bray-Curtis dissimilarity is a metric of beta diversity.

2.5 Supplementary Figure S5: Mann-Whitney U test statistic distributions from comparing Bray–Curtis dissimilarities within and between twin pairs

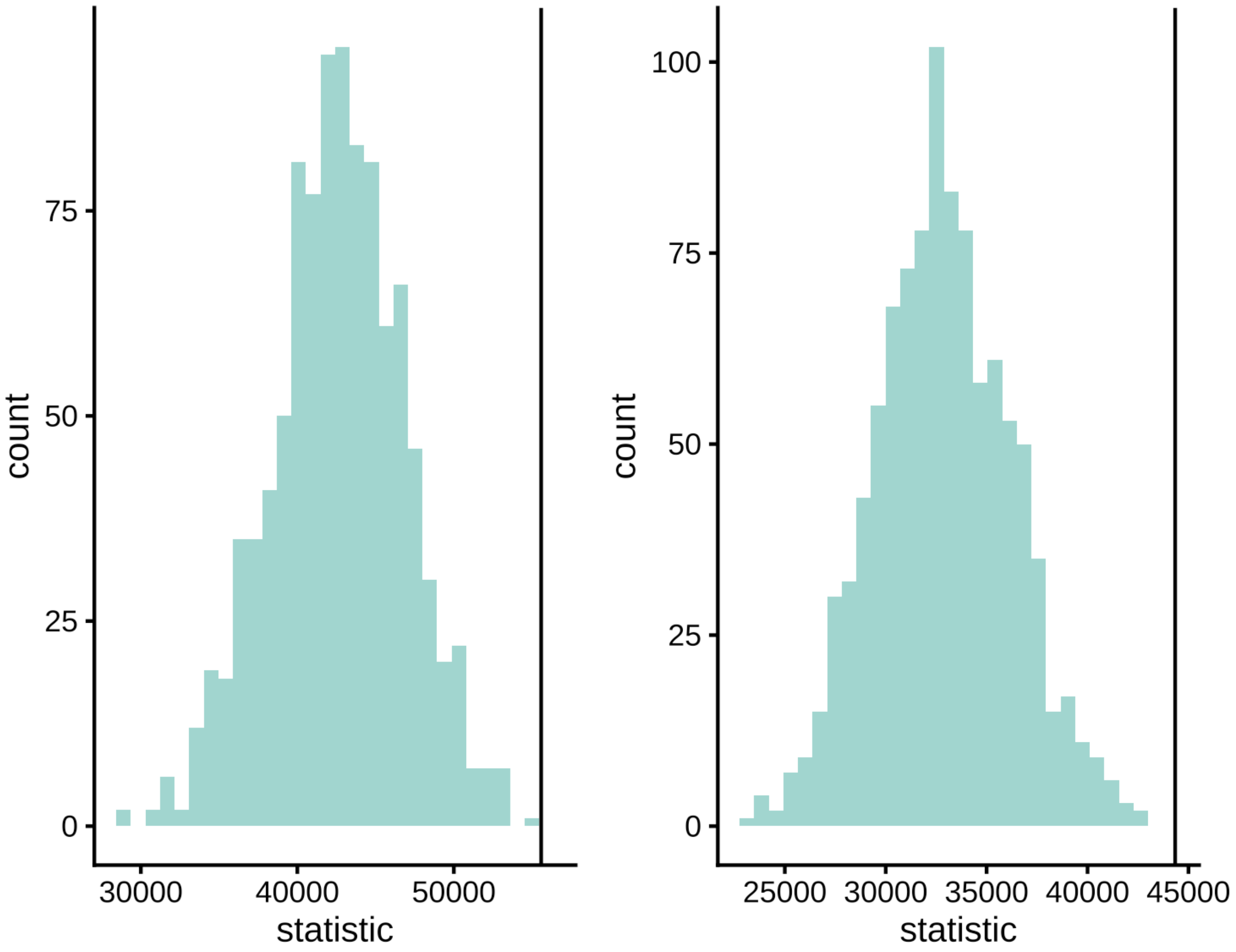

Original group labels (black line) and from 1,000 group label permutations (green). Left: Baseline, Right: End of study.

2.6 Supplementary Figure S6: Significant Microbiota Features between Baseline and Study End

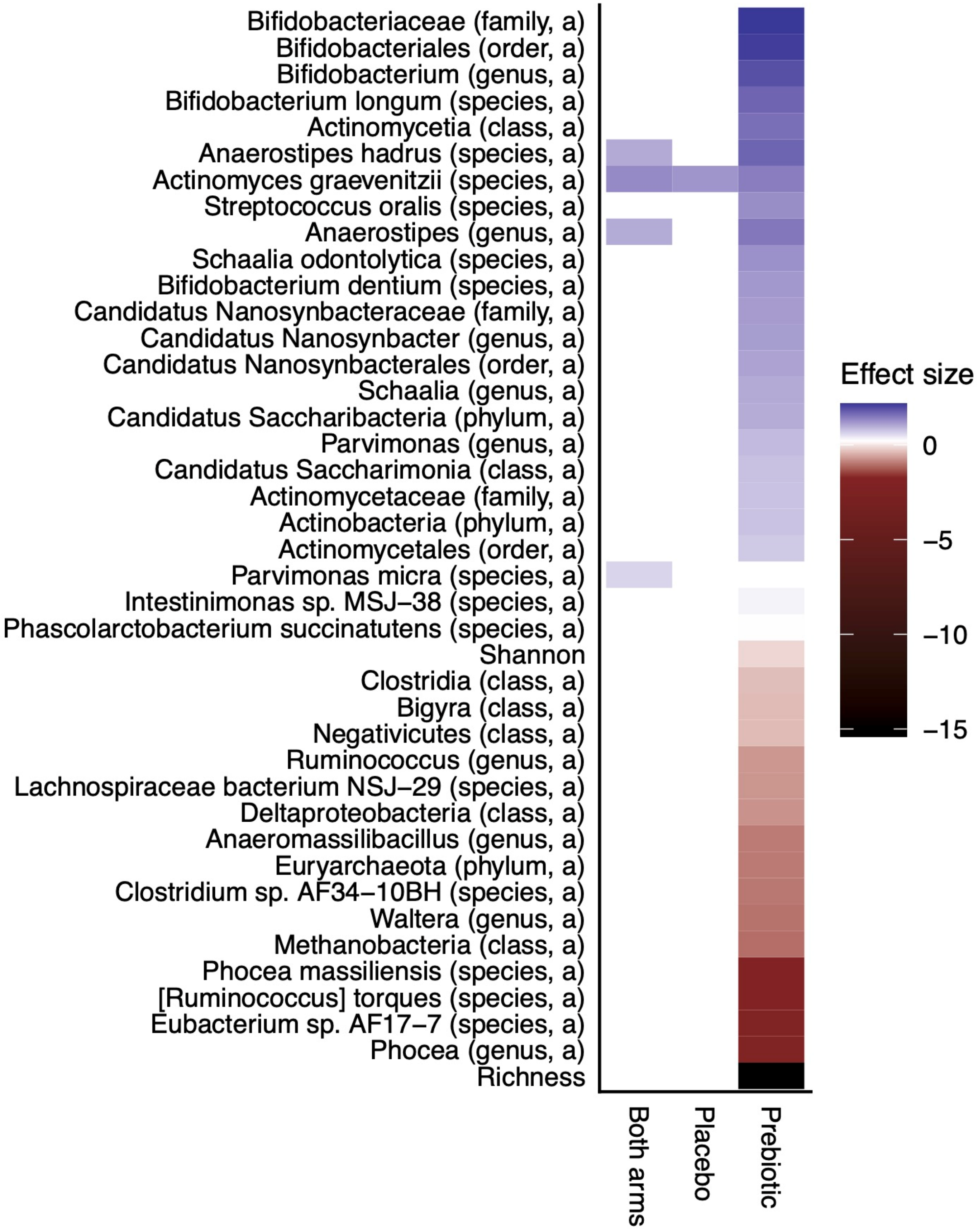

Paired group comparison of relative abundance (a) and prevalence (p) of bacterial taxa between baseline and end of study, including data from either study arm or both study arms. Negative effect sizes (blue) indicate features that a higher at study end compared to baseline while positive effect sizes (red) are lower at study end compared to

baseline. Effect sizes are the bias-corrected coefficients from the linear models, except for alpha diversity metrics which were not subjected to compositional bias correction.

2.7 Supplementary Figure S7: Intra-twin pair Bray-Curtis dissimilarity between visits

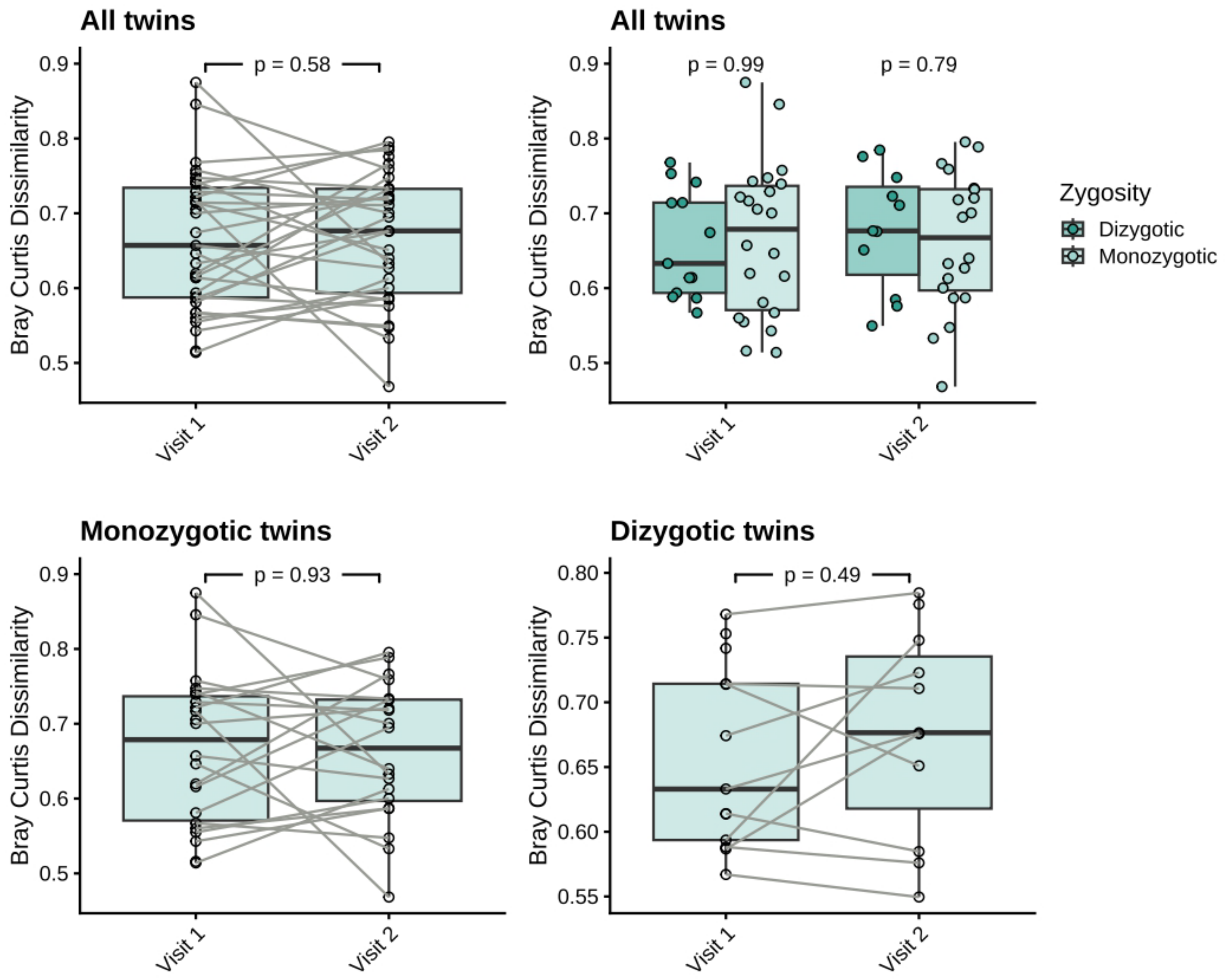

Intra-twin pair Bray-Curtis dissimilarity between visits (top left), visits by zygosity (top right), visits – monozygotic (bottom left), visits – dizygotic (bottom right)

2.8 *Supplementary Figure S8: Microbiota features significantly correlated with physical ability*

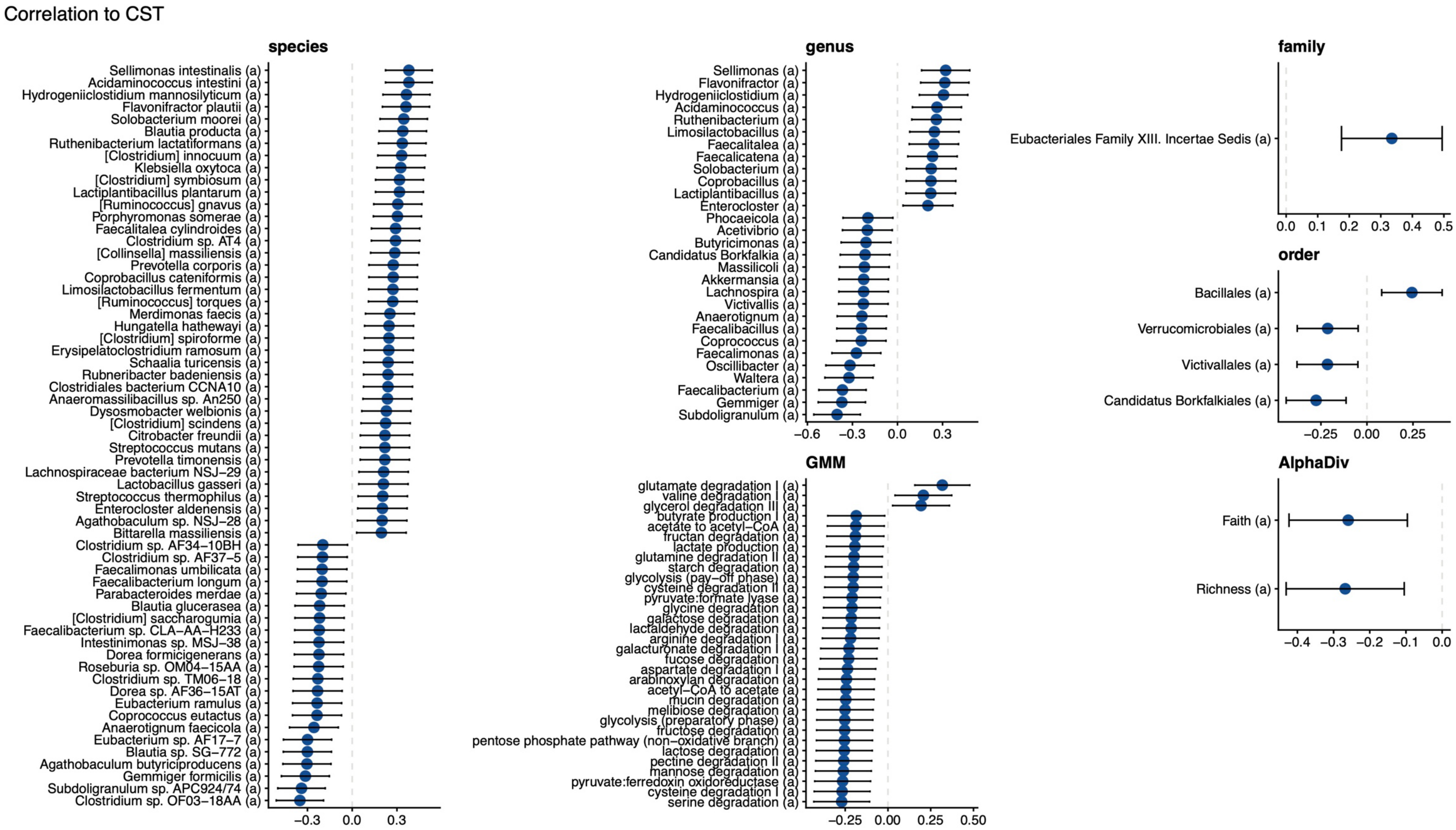

Partial Pearson correlations between physical ability (chair rise time) and microbiota feature abundance (a; blue dots) and prevalence (p; orange dots) adjusted for study arm allocation and twin pair. Correlation coefficients are calculated after bias-correction for taxonomical features (species, genus, family, order) while gut metabolic

modules (GMM), gut brain modules (GBM), and alpha diversity (AlphaDiv) coefficients have not been subjected to bias correction. CST: chair stand time (aka chair rise time).

2.9 *Supplementary Figure S9: Changes in Microbiota features significantly correlated with change in physical and cognition*

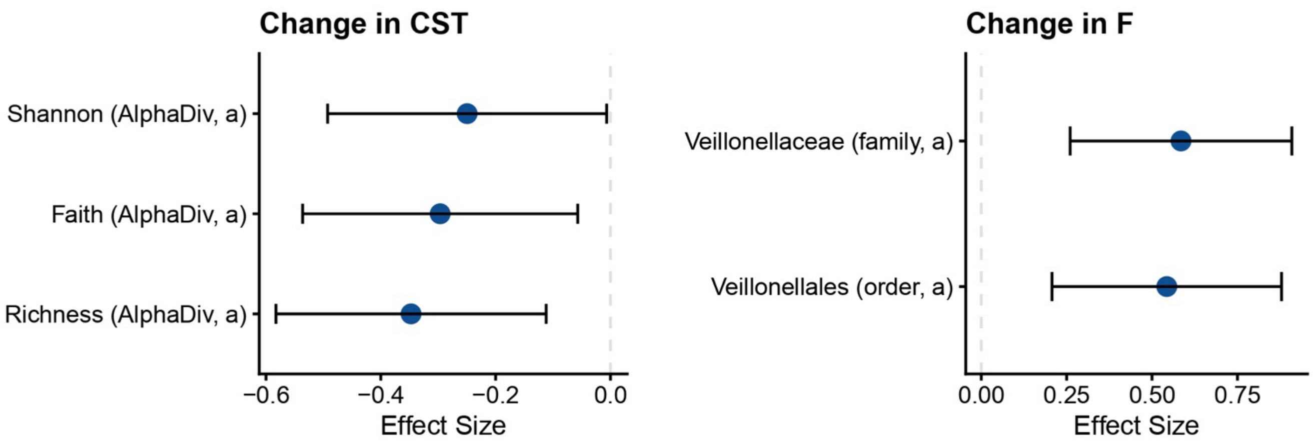

Partial Pearson correlations between the change in physical ability (chair rise time, CST; left) and cognition factor score (F, right) and microbiota feature abundance (blue dots) at study end adjusted for relative abundance at baseline, study arm, and twin pair. Change in chair rise time and cognition factor score were calculated as observations at study end minus observations at baseline. Correlation coefficients for taxa are calculated after bias-correction. AlphaDiv: alpha diversity.

2.10 Supplementary Figure S10: Microbiota features significantly correlated with cognition

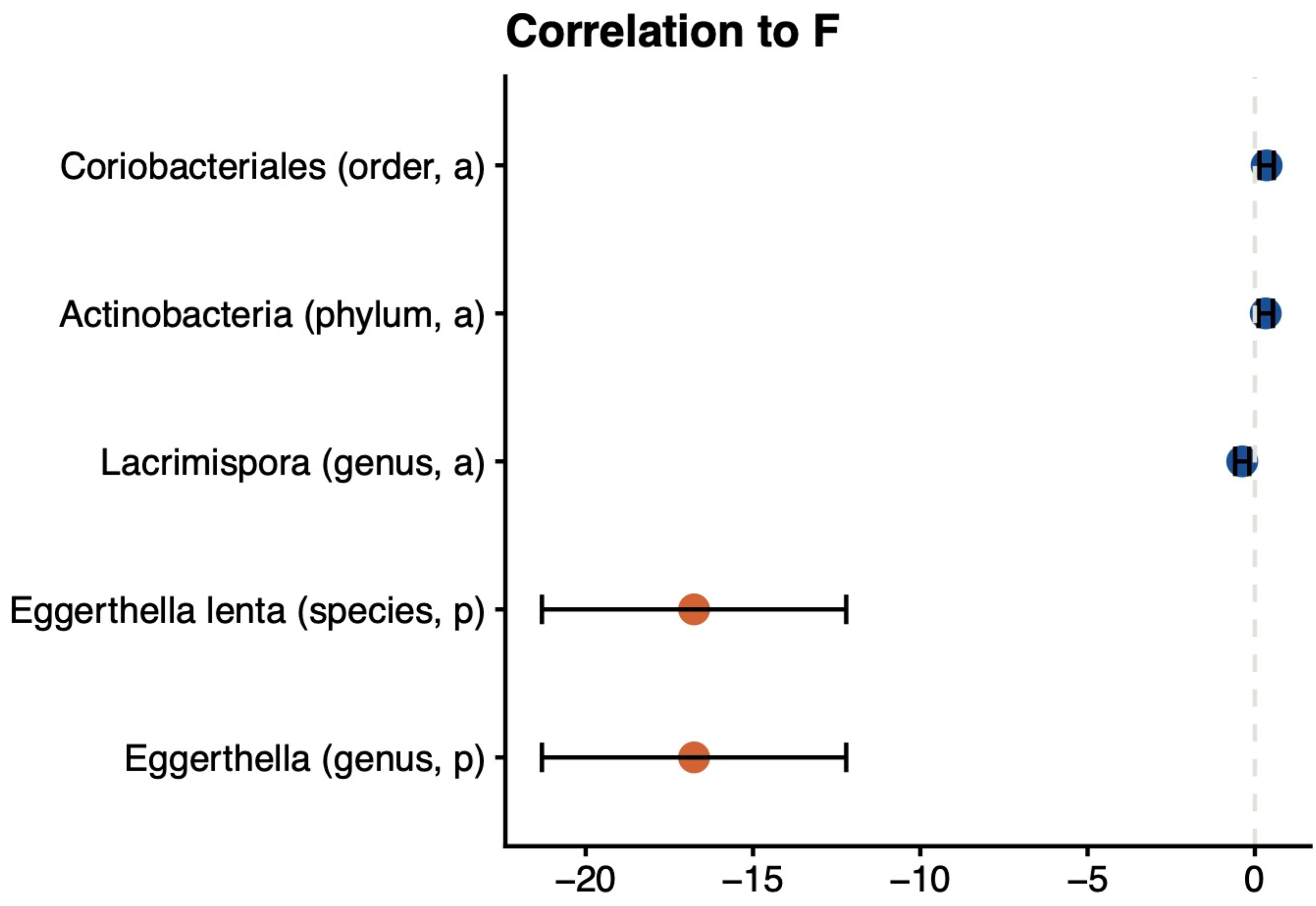

Partial Pearson correlations between cognitive ability (cognition factor score, F) and microbiota feature abundance (a; blue dots) and prevalence (p; orange dots) adjusted for study arm allocation and twin pair. Correlation coefficients are calculated after bias-correction.

3 Supplementary Tables

3.1 Supplementary Table S1: Demographic characteristics of those who declined or were ineligible to take part, versus those who took part.

		**Declined/Ineligible**	**Study Participant**	**p-value**
		N=525	N=72	
		Declined n=326<br>Ineligible n=199		
Age (years)		75 (5)	73 (5)	0.006*
Sex	Female	479 (91%)	56 (78%)	<0.001*
	Male	46 (9%)	16 (22%)	
Zygosity	Monozygotic	299 (57%)	44 (61%)	0.50
	Dizygotic	226 (43%)	28 (39%)	
Smoking Status	Never Smoked	299 (57%)	38 (53%)	0.48
	Ex-Smoker	206 (40%)	30 (42%)	
	Current Smoker	16 (3%)	4 (6%)	
Household Income	Declined to Answer	108 (23%)	13 (18%)	0.24
	Low Income	200 (43%)	30 (42%)	
	Middle Income	102 (22%)	23 (32%)	
	High Income	56 (12%)	6 (8%)	
Education Level	Low	239 (48%)	33 (46%)	0.95
	Middle	169 (34%)	25 (35%)	
	High	92 (18%)	14 (19%)	
Weight (kg)		71.9 (14.0)	73.8 (15.7)	0.31
Height (m)		1.6 (0.1)	1.6 (0.1)	0.64
Body Mass Index		27.2 (5.1)	28.0 (5.1)	0.22
Frailty Index		0.3 (0.1)	0.2 (0.1)	0.71

P values are group comparisons between each study arm performed using a two-sided paired t-test. Data for those who declined or were ineligible is the most recent available data in the longitudinal TwinsUK cohort. The difference between the two groups in terms of sex was adjusted for age, and likewise the difference between the two groups in terms of age was adjusted for sex, to assess whether one of these variables was driving all the variation between the two groups. However, a significant difference was retained in both cases. * Denotes significance.

3.2 Supplementary Table S2: Details of adverse events experienced by participants in the PROMOTe trial.

No.	Adverse Event	Arm	Related to Study?
1	Bloated, loss of appetite, waist feels bigger	2	probably
2	Bloating	1	probably
3	Bloating	1	probably
4	Flatulence*	1	probably
5	Wind	1	probably
6	Constipation	1	possibly
7	Diverticulitis	1	possibly
8	Cramps in legs	1	remote
9	Headaches	2	remote
10	Hair loss*	1	remote
11	Nose bleeds	1	remote

*Experienced by same participant. Arm 1 = prebiotic. Arm 2 = placebo.

3.3 Supplementary Table S3: Cognition Missingness

		Missing CANTAB at baseline	Not Missing CANTAB at baseline	p-value	Missing CANTAB at study end	Not missing CANTAB at study end	p-value
		N=22	N=50		N=46	N=26	
Age	Years	74.7 (5.0)	72.4 (4.6)	0.060	73.7 (5.2)	72.1 (4.0)	0.180
Sex	Female	20 (90.9%)	36 (72.0%)	0.075	37 (80.4%)	19 (73.1%)	0.470
	Male	2 (9.1%)	14 (28.0%)		9 (19.6%)	7 (26.9%)	
Zygosity	Monozygotic	11 (50.0%)	33 (66.0%)	0.200	28 (60.9%)	16 (61.5%)	0.960
	Dizygotic	11 (50.0%)	17 (34.0%)		18 (39.1%)	10 (38.5%)	
Smoking Status	Never Smoked	11 (50.0%)	27 (54.0%)	0.900	26 (56.5%)	12 (46.2%)	0.230
	Ex-Smoker	10 (45.5%)	20 (40.0%)		19 (41.3%)	11 (42.3%)	
	Current Smoker	1 (4.5%)	3 (6.0%)		1 (2.2%)	3 (11.5%)	
Household Income	Declined to Answer	3 (13.6%)	10 (20.0%)	0.920	11 (23.9%)	2 (7.7%)	0.170
	Low Income	10 (45.5%)	20 (40.0%)		18 (39.1%)	12 (46.2%)	
	Middle Income	7 (31.8%)	16 (32.0%)		12 (26.1%)	11 (42.3%)	
	High Income	2 (9.1%)	4 (8.0%)		5 (10.9%)	1 (3.8%)	
Education Level	Low	14 (63.6%)	19 (38.0%)	0.110	22 (47.8%)	11 (42.3%)	0.570
	Middle	6 (27.3%)	19 (38.0%)		14 (30.4%)	11 (42.3%)	
	High	2 (9.1%)	12 (24.0%)		10 (21.7%)	4 (15.4%)	
Alcohol Intake	Never Drink	4 (18.2%)	16 (32.0%)	0.370	11 (23.9%)	9 (34.6%)	0.420
	Less than weekly	7 (31.8%)	10 (20.0%)		10 (21.7%)	7 (26.9%)	
	At least weekly	11 (50.0%)	24 (48.0%)		25 (54.3%)	10 (38.5%)	
Body Mass Index	kg/m^2	27.3 (5.5)	28.3 (5.0)	0.420	27.7 (5.4)	28.6 (4.6)	0.480
Baseline Protein Intake	g/d	58.9 (15.5)	61.5 (13.9)	0.500	58.9 (16.2)	63.8 (9.9)	0.170
Baseline Protein/Weight	g/kg/d	0.9 (0.3)	0.8 (0.2)	0.620	0.8 (0.2)	0.9 (0.2)	0.300
Baseline Energy Intake	Kcals/d	1539.5 (288.7)	1582.7 (439.1)	0.670	1550.7 (405.8)	1602.8 (388.4)	0.600
Frailty Index		0.3 (0.2)	0.2 (0.1)	0.210	0.2 (0.1)	0.2 (0.1)	0.980
Baseline Gait speed	m/sec	1.2 (0.3)	1.1 (0.4)	0.380	1.1 (0.4)	1.1 (0.3)	0.430
Baseline Chair Rise Time	sec	10.7 (4.4)	10.6 (2.8)	0.930	10.9 (3.5)	10.0 (2.9)	0.270

Baseline Grip Strength	kg	24.0 (10.3)	26.9 (9.4)	0.250	26.5 (10.4)	25.0 (8.5)	0.520
Baseline Balance Score		4.0 (0.2)	4.0 (0.1)	0.550	4.0 (0.1)	4.0 (0.2)	0.680
Baseline SPPB Score		10.9 (1.8)	11.0 (1.3)	0.640	10.9 (1.6)	11.2 (1.3)	0.380
Baseline IPAQ	MET mins/week	730.6 (756.9)	650.2 (610.8)	0.630	674.2 (645.4)	675.9 (683.2)	0.990
Baseline IPAQ Score	1	21 (95.5%)	48 (96.0%)	0.920	45 (98%)	24 (92%)	0.260
	2	1 (4.5%)	2 (4.0%)		1 (2%)	2 (8%)	
Baseline SNAQ Score		14.8 (2.3)	15.4 (1.9)	0.250	15.2 (2.1)	15.3 (1.9)	0.870
Healthy Eating Index		60.8 (8.0)	59.1 (11.5)	0.550	61.4 (10.3)	56.9 (11.0)	0.099

CANTAB: Cambridge Neuropsychological Test Automated Battery. SPPB: Short Physical Performance Battery. IPAQ: International Physical Activity Questionnaire. MET: metabolic equivalent of task (One MET is the energy you expend at rest). SNAQ: Simplified Nutritional Assessment Questionnaire. P values are within group comparisons of the total population between baseline and end of intervention performed using a two-sided paired t-test for continuous data and Pearson’s chi squared test for categorical data. Analyses were not adjusted for multiple testing.

3.4 Supplementary Table S4: PROMOTe Dietary Intakes from Background Diet at Baseline and End time points.

	**Baseline**				**End**			
**Dietary variable**	**Females**	**Males**	**Total**		**Females**	**Males**	**Total**	**P-value**
**Energy, macronutrients, components**	N=56	N=16	N=72		N=56	N=16	N=72	
Energy kcal/d	1504.4 (347.6)	1797.5 (484.0)	1569.5 (397.7)		1410.6 (383.4)	1672.4 (546.4)	1476.0 (438.7)	0.021*
Protein g/d	58.2 (15.6)	67.5 (11.9)	60.2 (15.2)		59.2 (18.0)	55.0 (20.2)	58.2 (18.4)	0.321
Fat g/d	63.0 (19.3)	76.9 (26.2)	66.1 (21.6)		59.9 (20.1)	70.9 (26.4)	62.7 (22.1)	0.032*
Cholesterol g/d	236.2 (106.1)	255.0 (108.4)	240.3 (106.0)		253.4 (123.7)	178.7 (136.6)	234.7 (129.7)	0.676
Carbohydrate g/d	157.4 (56.8)	199.8 (59.9)	166.7 (59.6)		149.2 (55.1)	185.7 (60.5)	158.4 (58.1)	0.115
Glucose g/d	13.2 (8.2)	13.3 (8.1)	13.2 (8.1)		13.0 (8.8)	12.5 (5.4)	12.8 (8.1)	0.221
Sugar g/d	71.5 (34.5)	82.8 (47.1)	74.0 (37.5)		65.5 (31.3)	82.3 (43.0)	69.7 (34.9)	0.106
Oligosaccharide g/d	0.9 (1.8)	1.0 (1.3)	0.9 (1.7)		0.4 (0.6)	0.4 (0.7)	0.4 (0.6)	0.329
Starch g/d	81.9 (34.9)	115.9 (19.1)	89.4 (34.9)		80.5 (34.2)	94.8 (33.3)	84.1 (34.2)	0.186
Fibre AOAC, g/d	16.5 (5.9)	15.9 (5.7)	16.3 (5.8)		15.5 (6.2)	18.9 (7.6)	16.3 (6.7)	0.951
Fibre NSP, g/d	11.6 (4.1)	11.0 (3.6)	11.4 (4.0)		11.3 (4.9)	12.2 (4.7)	11.5 (4.8)	0.697
**Minerals**								
Sodium mg/d	1723.5 (763.8)	2312.9 (752.6)	1853.4 (794.0)		1727.0 (640.7)	1698.9 (698.2)	1720.0 (648.0)	0.341
Potassium mg/d	2384.2 (627.6)	2670.0 (874.7)	2447.2 (691.5)		2252.0 (731.7)	2649.0 (911.3)	2351.2 (789.5)	0.099
Calcium mg/d	684.5 (258.1)	900.9 (272.2)	732.2 (274.2)		652.8 (389.6)	724.2 (331.5)	670.7 (373.8)	0.370
Magnesium mg/d	226.0 (66.7)	266.2 (82.7)	234.8 (71.8)		213.0 (78.2)	281.2 (109.6)	230.0 (90.8)	0.466
Iron mg/d	8.9 (2.4)	8.8 (1.9)	8.9 (2.3)		8.3 (3.7)	9.1 (4.5)	8.5 (3.9)	0.892
Zinc mg/d	6.7 (2.2)	7.9 (1.9)	7.0 (2.2)		6.7 (2.5)	6.7 (2.7)	6.7 (2.6)	0.142
Selenium µg/d	37.8 (15.8)	41.9 (17.6)	38.7 (16.1)		37.6 (16.5)	39.3 (21.9)	38.0 (17.7)	0.998

**Vitamins**								
Vitamin A µg/d	0.0 (0.0)	0.0 (0.0)	0.0 (0.0)		0.0 (0.0)	0.0 (0.0)	0.0 (0.0)	-
Thiamine mg/d	1.2 (0.4)	1.3 (0.4)	1.2 (0.4)		1.3 (1.3)	1.2 (0.4)	1.3 (1.1)	0.519
Riboflavin mg/d	1.3 (0.5)	1.5 (0.5)	1.3 (0.5)		1.3 (0.7)	1.3 (0.6)	1.3 (0.6)	0.132
Niacin mg/d	14.5 (5.0)	15.0 (6.0)	14.6 (5.2)		15.1 (7.0)	16.5 (6.9)	15.5 (6.9)	0.609
Vitamin B6 µ/d	1.5 (0.5)	1.6 (0.6)	1.5 (0.5)		1.4 (0.5)	1.6 (0.6)	1.4 (0.6)	0.188
Vitamin B12 µg/d	3.5 (1.9)	4.4 (2.3)	3.7 (2.0)		2.9 (1.6)	2.6 (1.3)	2.8 (1.5)	0.026*
Folate µg/d	222.1 (82.9)	212.5 (77.3)	220.0 (81.2)		183.8 (77.1)	212.8 (99.9)	191.1 (83.2)	0.079
Vitamin C µg/d	91.4 (73.9)	61.9 (26.1)	84.9 (67.3)		69.9 (47.3)	72.4 (43.5)	70.5 (46.0)	0.084
Vitamin D µg/d	3.5 (3.8)	2.9 (1.9)	3.3 (3.4)		2.7 (2.3)	2.1 (2.8)	2.5 (2.4)	0.200
25-Hydroxy D3 µg/d	0.0 (0.1)	0.0 (0.0)	0.0 (0.1)		0.4 (1.2)	0.0 (0.2)	0.3 (1.0)	0.092
Vitamin D3 µg/d	0.3 (0.6)	0.6 (0.9)	0.4 (0.7)		0.3 (0.7)	0.3 (0.8)	0.3 (0.7)	0.228
Vitamin E µg/d	8.0 (3.3)	8.9 (5.8)	8.2 (3.9)		6.2 (2.6)	8.2 (5.1)	6.9 (3.4)	0.014*
Vitamin K1 µg/d	78.3 (89.5)	54.7 (37.4)	73.1 (81.3)		50.2 (37.8)	49.1 (53.5)	49.9 (41.6)	0.442
**Food groups and other**								
Vegetables g/d	160 (120)	107 (63)	149 (112)		141 (64)	95 (61)	129 (66)	0.469
Fruits g/d	143.5 (104.2)	148.9 (107.7)	144.7 (104.0)		141.2 (141.5)	143.1 (92.2)	141.7 (130.0)	0.167
Alcohol g/d	9.7 (15.6)	18.6 (21.3)	11.6 (17.2)		7.7 (8.7)	13.5 (19.8)	9.1 (12.5)	0.063
Water g/d	1931.3 (625.8)	2075.4 (840.2)	1963.0 (673.4)		1858.9 (622.3)	1848.2 (782.2)	1856.2 (657.0)	0.124
Missing Data (n)	10	3	13		20	4	24	

All data presented as mean (standard deviation). Please note the nutritional intake from the intervention sachet is not included in these dietary data. NSP: non-starch polysaccharides. AOAC fibre comprises of the total amount of non-digestible polysaccharides, and includes e.g., lignin and resistant starches, measured with a set of methods developed by the Association of Analytical Chemists (AOAC). * Denotes significance.
P values are within group comparisons of the total population between baseline and end of intervention performed using a two-sided paired t-test

3.5 *Supplementary Table S5: PROMOTe Dietary Intakes from Background Diet at Baseline and End time points, with Over Reporters removed.*

	**Baseline**			**End**			
**Dietary variable**	**Females**	**Males**	**Total**	**Females**	**Males**	**Total**	**P-value**
**Energy, macronutrients, components**	N=54	N=15	N=69	N=55	N=15	N=70	
Energy kcal/d	1528.1 (330.0)	1824.8 (488.1)	1592.6 (386.2)	1431.8 (366.9)	1773.7 (439.4)	1513.5 (407.8)	0.0100*
Protein g/d	59.7 (14.0)	67.2 (12.3)	61.3 (13.9)	59.7 (18.1)	58.4 (17.2)	59.4 (17.7)	0.1989
Fat g/d	64.9 (17.3)	79.4 (25.7)	68.0 (20.1)	61.0 (19.4)	74.8 (23.8)	64.3 (21.1)	0.0163*
Cholesterol g/d	240.2 (106.3)	242.3 (102.7)	240.7 (104.7)	256.2 (124.3)	191.9 (134.9)	240.8 (128.4)	0.6742
Carbohydrate g/d	161.1 (55.1)	205.3 (58.9)	170.5 (58.4)	151.4 (54.4)	197.0 (48.4)	162.3 (56.1)	0.1363
Glucose g/d	13.4 (8.3)	13.5 (8.5)	13.4 (8.2)	13.2 (8.8)	13.2 (5.0)	13.2 (8.0)	0.2216
Sugar g/d	72.8 (34.6)	85.6 (48.1)	75.5 (37.7)	66.5 (31.1)	87.4 (41.1)	71.5 (34.5)	0.0981
Oligosaccharide g/d	0.9 (1.8)	1.0 (1.3)	1.0 (1.7)	0.4 (0.6)	0.5 (0.7)	0.4 (0.6)	0.3189
Starch g/d	84.2 (33.9)	118.8 (16.7)	91.6 (34.1)	81.5 (34.1)	101.3 (25.7)	86.2 (33.1)	0.2676
Fibre AOAC, g/d	16.9 (5.6)	16.1 (5.9)	16.7 (5.6)	15.6 (6.2)	20.2 (6.6)	16.7 (6.5)	0.9588
Fibre NSP, g/d	11.9 (3.9)	11.2 (3.7)	11.7 (3.9)	11.4 (4.9)	13.1 (3.8)	11.8 (4.7)	0.8229
**Minerals**							
Sodium mg/d	1770.8 (746.1)	2346.3 (775.9)	1894.1 (782.6)	1738.8 (646.1)	1828.8 (560.0)	1760.3 (621.8)	0.4561
Potassium mg/d	2424.1 (607.9)	2647.5 (909.7)	2472.0 (680.4)	2271.7 (732.5)	2836.0 (672.4)	2406.7 (751.8)	0.0908

Calcium mg/d	704.0 (246.6)	928.3 (264.9)	752.1 (264.9)		661.3 (392.0)	786.2 (264.9)	691.1 (366.8)	0.4012
Magnesium mg/d	229.6 (65.8)	267.9 (86.1)	237.8 (71.5)		215.5 (77.8)	298.8 (95.5)	235.4 (88.8)	0.4881
Iron mg/d	9.0 (2.2)	8.9 (2.0)	9.0 (2.2)		8.4 (3.7)	9.7 (4.2)	8.7 (3.8)	0.9016
Zinc mg/d	6.9 (2.0)	7.9 (1.9)	7.1 (2.0)		6.7 (2.5)	7.2 (2.3)	6.9 (2.4)	0.1627
Selenium μg/d	38.5 (15.7)	39.0 (14.9)	38.6 (15.4)		38.2 (16.3)	34.7 (15.7)	37.4 (16.0)	0.9063
**Vitamins**								
Vitamin A μg/d	0.0 (0.0)	0.0 (0.0)	0.0 (0.0)		0.0 (0.0)	0.0 (0.0)	0.0 (0.0)	-
Thiamine mg/d	1.2 (0.3)	1.3 (0.5)	1.2 (0.4)		1.4 (1.3)	1.3 (0.3)	1.4 (1.1)	0.5558
Riboflavin mg/d	1.3 (0.4)	1.5 (0.5)	1.4 (0.5)		1.3 (0.7)	1.4 (0.5)	1.3 (0.6)	0.2108
Niacin mg/d	14.8 (4.8)	13.9 (4.8)	14.6 (4.8)		15.2 (7.1)	16.2 (7.2)	15.4 (7.0)	0.8858
Vitamin B6 μ/d	1.5 (0.4)	1.6 (0.6)	1.5 (0.5)		1.4 (0.6)	1.7 (0.5)	1.5 (0.5)	0.1624
Vitamin B12 μg/d	3.6 (1.8)	4.0 (2.0)	3.7 (1.9)		2.9 (1.6)	2.6 (1.4)	2.8 (1.6)	0.0517
Folate μg/d	228.6 (78.8)	203.5 (73.3)	223.2 (77.7)		185.3 (77.7)	230.5 (82.8)	196.1 (80.4)	0.0902
Vitamin C μg/d	93.1 (75.1)	62.3 (27.2)	86.5 (68.7)		71.2 (47.3)	77.1 (42.5)	72.6 (45.8)	0.0495*
Vitamin D μg/d	3.6 (3.8)	2.6 (1.6)	3.4 (3.5)		2.7 (2.4)	2.0 (2.9)	2.5 (2.5)	0.1731
25-Hydroxy D3 μg/d	0.0 (0.1)	0.0 (0.0)	0.0 (0.1)		0.4 (1.2)	0.1 (0.2)	0.3 (1.1)	0.0904
Vitamin D3 μg/d	0.3 (0.6)	0.5 (0.8)	0.4 (0.7)		0.3 (0.7)	0.1 (0.4)	0.3 (0.6)	0.0982
Vitamin E μg/d	8.2 (3.2)	9.3 (5.9)	8.4 (3.9)		6.3 (2.6)	8.7 (5.0)	6.9 (3.4)	0.0153*
Vitamin K1 μg/d	80.7 (90.8)	54.2 (39.0)	75.0 (82.9)		51.4 (37.6)	53.0 (54.3)	51.8 (41.5)	0.5882

**Food groups and other**								
Vegetables g/d	161.9 (122.2)	110.3 (64.9)	150.8 (113.9)		143.2 (63.4)	103.2 (55.8)	133.6 (63.4)	0.5520
Fruits g/d	145.5 (105.6)	152.4 (111.7)	147.0 (105.9)		142.6 (143.3)	154.4 (87.5)	145.4 (131.3)	0.1022
Alcohol g/d	8.2 (13.1)	18.0 (22.2)	10.3 (15.8)		7.9 (8.8)	14.7 (20.3)	9.5 (12.6)	0.0182*
Water g/d	1956.7 (626.8)	2023.9 (855.8)	1971.1 (674.1)		1865.7 (630.1)	1975.9 (676.8)	1892.0 (635.6)	0.0939
Missing Data (n)	11	3	14		20	4	24	

All data presented as mean (standard deviation). NSP: non-starch polysaccharides. AOAC fibre comprises of the total amount of non-digestible polysaccharides, and includes e.g., lignin and resistant starches, measured with a set of methods developed by the Association of Analytical Chemists (AOAC). Please note the nutritional intake from the intervention sachet is not included in these dietary data. * Denotes significance.
P values are within group comparisons of the total population between baseline and end of intervention performed using a two-sided paired t-test.

3.6 Supplementary Table S6: PROMOTe Dietary Intakes from Background Diet and weight/BMI, by Study Arm

**Dietary variable**	**Baseline**			**End**			**Baseline v End Prebiotic**	**Baseline v End Placebo**
	**Prebiotic arm**	**Placebo**	**p-value**	**Prebiotic arm**	**Placebo**	**p-value**	**p-value**	**p-value**
	N=36	N=36		N=36	N=36			
**Energy, macronutrients, components**								
Energy kcals/d	1581.6 (380.5)	1557.5 (419.2)	0.8	1449.2 (339.6)	1500.7 (519.5)	0.69	0.01*	0.30
Protein g/d	60.5 (17.6)	60.0 (12.6)	0.91	58.0 (14.9)	58.3 (21.5)	0.95	0.44	0.53
Fat g/d	70.4 (22.5)	61.5 (19.9)	0.11	61.6 (18.6)	63.6 (25.3)	0.75	0.01*	0.60
Cholesterol g/d	243.4 (107.7)	237.1 (105.9)	0.82	238.7 (135.6)	231.1 (126.8)	0.84	0.82	0.26
Carbohydrate g/d	162.0 (52.9)	171.6 (66.5)	0.54	155.0 (43.7)	161.4 (69.5)	0.71	0.06	0.56
Glucose g/d	13.0 (8.9)	13.4 (7.3)	0.84	12.6 (7.8)	13.1 (8.5)	0.83	0.11	0.87
Sugar g/d	71.6 (29.8)	76.5 (44.5)	0.61	67.4 (29.8)	71.8 (39.4)	0.67	0.09	0.51
Oligosaccharide g/d	1.0 (2.0)	0.9 (1.3)	0.88	0.4 (0.5)	0.4 (0.6)	0.69	0.90	0.23
Starch g/d	86.3 (34.0)	92.7 (36.2)	0.48	83.2 (30.5)	84.9 (37.8)	0.87	0.15	0.57
Fibre AOAC g/d	17.0 (6.7)	15.6 (4.7)	0.34	16.1 (4.8)	16.6 (8.1)	0.8	0.33	0.46
Fibre NSP g/d	12.0 (4.7)	10.8 (3.0)	0.25	11.8 (3.8)	11.2 (5.6)	0.65	0.13	0.68

**Minerals**								
Sodium mg/d	1927.7 (945.2)	1776.6 (607.2)	0.47	1737.1 (435.4)	1704.2 (805.0)	0.86	0.20	0.996
Potassium mg/d	2493.0 (704.8)	2399.7 (686.7)	0.61	2392.7 (597.9)	2313.1 (943.2)	0.73	0.05	0.50
Calcium mg/d	765.4 (319.8)	697.9 (217.8)	0.35	676.1 (239.8)	665.7 (470.0)	0.92	0.07	0.88
Magnesium mg/d	247.7 (75.6)	221.5 (66.3)	0.16	226.6 (72.6)	233.1 (106.3)	0.81	0.04*	0.58
Iron mg/d	9.0 (2.4)	8.7 (2.1)	0.64	7.8 (2.0)	9.2 (5.0)	0.22	0.06	0.54
Zinc mg/d	6.9 (2.4)	7.0 (2.0)	0.89	6.7 (2.3)	6.7 (2.8)	0.97	0.38	0.26
Selenium µg/d	39.7 (17.2)	37.7 (15.1)	0.63	34.5 (16.4)	41.2 (18.6)	0.19	0.73	0.80
**Vitamins**								
Vitamin A µg/d	0.0 (0.0)	0.0 (0.0)		0.0 (0.0)	0.0 (0.0)			
Thiamine mg/d	1.2 (0.3)	1.2 (0.4)	0.51	1.2 (0.3)	1.5 (1.5)	0.37	0.48	0.73
Riboflavin mg/d	1.4 (0.5)	1.3 (0.5)	0.78	1.2 (0.4)	1.3 (0.8)	0.86	0.23	0.34
Niacin mg/d	14.4 (5.0)	14.9 (5.5)	0.71	14.4 (5.8)	16.4 (7.8)	0.31	0.82	0.39
Vitamin B6 µg/d	1.4 (0.4)	1.6 (0.6)	0.25	1.4 (0.3)	1.4 (0.7)	0.99	0.41	0.31
Vitamin B12 µg/d	3.7 (1.9)	3.6 (2.1)	0.78	2.8 (1.5)	2.8 (1.6)	0.96	0.54	<0.01*
Folate µg/d	221.6 (80.4)	218.3 (83.3)	0.88	200.3 (59.9)	182.6 (100.5)	0.47	0.08	0.24
Vitamin C µg/d	89.2 (70.3)	80.4 (65.1)	0.62	74.1 (46.0)	67.2 (46.6)	0.61	0.18	0.24
Vitamin D µg/d	3.6 (4.1)	3.0 (2.6)	0.51	2.6 (2.6)	2.4 (2.3)	0.7	0.92	0.05

25-Hydroxy Vitamin D3 µg/d	0.0 (0.1)	0.0 (0.0)	0.32	0.4 (1.3)	0.2 (0.7)	0.46	0.20	0.25
Vitamin D3 µg/d	0.3 (0.5)	0.5 (0.9)	0.19	0.2 (0.6)	0.4 (0.8)	0.34	0.96	0.15
Vitamin E µg/d	9.0 (4.1)	7.4 (3.7)	0.13	6.5 (3.0)	6.9 (3.9)	0.7	0.04*	0.21
Vitamin K1 µg/d	75.1 (92.2)	71.0 (69.8)	0.85	36.8 (36.5)	62.0 (43.0)	0.035	0.35	0.79
**Food groups and other**								
Vegetables g/d	156.5 (129.5)	140.4 (91.5)	0.59	130.0 (62.2)	128.9 (69.9)	0.95	0.81	0.50
Fruit g/d	141.8 (103.4)	147.7 (106.4)	0.83	143.0 (125.3)	140.5 (136.7)	0.95	0.28	0.40
Alcohol g/d	11.4 (16.6)	11.9 (18.1)	0.91	9.1 (14.1)	9.2 (11.0)	0.97	0.06	0.35
Water g/d	2044.8 (681.6)	1878.4 (666.1)	0.35	1958.8 (568.1)	1761.8 (728.0)	0.3	0.57	0.12
**Weight/BMI**								
Weight (kg)	71.8 (14.0)	75.8 (17.2)	0.28	72.7 (13.7)	75.0 (15.5)	0.54	0.58	0.68
Body mass index	27.3 (5.0)	28.7 (5.3)	0.24	27.7 (4.9)	28.3 (4.2)	0.57	0.51	0.58

All data presented as mean (standard deviation). NSP: non-starch polysaccharides. AOAC fibre comprises of the total amount of non-digestible polysaccharides, and includes e.g., lignin and resistant starches, measured with a set of methods developed by the Association of Analytical Chemists (AOAC). Please note the nutritional intake from the intervention sachet is not included in these dietary data. *Denotes statistical significance.

Going from left to right, P values are comparisons between prebiotic and placebo groups at baseline, between prebiotic and placebo groups at study end, within group comparison between baseline and study end for prebiotic arm, and within group comparison between baseline and study end for placebo arm, performed using a two-sided paired t-test.

3.7 Supplementary Table S7: Bowel Habit Questionnaire Responses

		Time Point		Total	Prebiotic	Placebo	p-value		Time Point		Total	Prebiotic	Placebo	p-value
				N=66	N=34	N=32					N=66	N=34	N=32	
Have you experienced any of the following in the last 4 weeks?														
	Diarrhoea	Baseline	No	50 (75.8%)	25 (73.5%)	25 (78.1%)	0.32		Study End	No	51 (77.3%)	27 (79.4%)	24 (75.0%)	0.52
			Yes	1 (1.5%)	1 (2.9%)	0 (0.0%)				Yes	6 (9.1%)	4 (11.8%)	2 (6.2%)	
			Missing	15 (22.7%)	8 (23.5%)	7 (21.9%)				Missing	9 (13.6%)	3 (8.8%)	6 (18.8%)	
	Constipation	Baseline	No	43 (65.2%)	23 (67.6%)	20 (62.5%)	0.47		Study End	No	45 (68.2%)	25 (73.5%)	20 (62.5%)	0.75
			Yes	12 (18.2%)	5 (14.7%)	7 (21.9%)				Yes	10 (15.2%)	5 (14.7%)	5 (15.6%)	
			Missing	11 (16.7%)	6 (17.6%)	5 (15.6%)				Missing	11 (16.7%)	4 (11.8%)	7 (21.9%)	
Stool frequency over previous 7 days														
		Baseline	2-3	2 (3.0%)	1 (2.9%)	1 (3.1%)	0.87		Study End	2-3	4 (6.1%)	2 (5.9%)	2 (6.2%)	0.67

			4-6	23 (34.8%)	11 (32.4%)	12 (37.5%)				4-6	23 (34.8%)	11 (32.4%)	12 (37.5%)	
			7 or more	29 (43.9%)	16 (47.1%)	13 (40.6%)				7 or more	30 (45.5%)	18 (52.9%)	12 (37.5%)	
			Missing	12 (18.2%)	6 (17.6%)	6 (18.8%)				Missing	9 (13.6%)	3 (8.8%)	6 (18.8%)	
Has the frequency of your bowel movements changed in the last 12 weeks/3 months?														
		Baseline	No	51 (77.3%)	26 (76.5%)	25 (78.1%)	0.6		Study End	No	47 (71.2%)	24 (70.6%)	23 (71.9%)	0.27
			Yes	3 (4.5%)	2 (5.9%)	1 (3.1%)				Yes	10 (15.2%)	7 (20.6%)	3 (9.4%)	
			Missing	12 (18.2%)	6 (17.6%)	6 (18.8%)				Missing	9 (13.6%)	3 (8.8%)	6 (18.8%)	
Using the Stool Chart provided, please define the consistency of your stool on average in the last 12 weeks/3 months.														
	Standard Bristol Stool Form Scale	Baseline	1	4 (6.1%)	2 (5.9%)	2 (6.2%)	0.6		Study End	1	1 (1.5%)	1 (2.9%)	0 (0.0%)	0.4
			2	2 (3.0%)	1 (2.9%)	1 (3.1%)				2	8 (12.1%)	6 (17.6%)	2 (6.2%)	
			3	19 (28.8%)	10 (29.4%)	9 (28.1%)				3	19 (28.8%)	8 (23.5%)	11 (34.4%)	

			4	17 (25.8%)	10 (29.4%)	7 (21.9%)				4	22 (33.3%)	13 (38.2%)	9 (28.1%)	
			5	2 (3.0%)	1 (2.9%)	1 (3.1%)				5	4 (6.1%)	1 (2.9%)	3 (9.4%)	
			6	3 (4.5%)	0 (0.0%)	3 (9.4%)				6	3 (4.5%)	2 (5.9%)	1 (3.1%)	
			7	1 (1.5%)	0 (0.0%)	1 (3.1%)				7	0 (0.0%)	0 (0.0%)	0 (0.0%)	
			Missing	18 (27.3%)	10 (29.4%)	8 (25.0%)				Missing	9 (13.6%)	3 (8.8%)	6 (18.8%)	

P values are within group comparisons of the total population between baseline and end of intervention performed using a two-sided paired t-test.

3.8 Supplementary Table S8: Average, minimum and maximum values of quality control and read mapping statistics for microbiome data

	Min	Average	Max
Read pairs	11.7 M	21.6 M	57.3 M
High quality reads	11.7 M	21.6 M	57.3 M
High quality non-host reads	11.7 M	21.5 M	57.3 M
Mapped to gene catalogue	8.8 M	17.9 M	48.8 M
Gene catalogue representation	68.5 %	82.8 %	88.3 %

References:

1. Pasolli, E. *et al.* Extensive Unexplored Human Microbiome Diversity Revealed by Over 150,000 Genomes from Metagenomes Spanning Age, Geography, and Lifestyle. *Cell* **176**, 649-662.e20 (2019).
2. Nielsen, H. B. *et al.* Identification and assembly of genomes and genetic elements in complex metagenomic samples without using reference genomes. *Nat Biotechnol* **32**, 822–828 (2014).
3. Parks, D. H., Imelfort, M., Skennerton, C. T., Hugenholtz, P. & Tyson, G. W. CheckM: assessing the quality of microbial genomes recovered from isolates, single cells, and metagenomes. *Genome Res* **25**, 1043–1055 (2015).
4. Langmead, B. & Salzberg, S. L. Fast gapped-read alignment with Bowtie 2. *Nat Methods* **9**, 357–359 (2012).
5. Schubert, M., Lindgreen, S. & Orlando, L. AdapterRemoval v2: rapid adapter trimming, identification, and read merging. *BMC Res Notes* **9**, 88 (2016).
6. Li, H. & Durbin, R. Fast and accurate short read alignment with Burrows–Wheeler transform. *Bioinformatics* **25**, 1754–1760 (2009).
7. Huerta-Cepas, J. *et al.* Fast Genome-Wide Functional Annotation through Orthology Assignment by eggNOG-Mapper. *Mol Biol Evol* **34**, 2115–2122 (2017).
8. Kanehisa, M. KEGG: Kyoto Encyclopedia of Genes and Genomes. *Nucleic Acids Res* **28**, 27–30 (2000).
9. Vieira-Silva, S. *et al.* Species–function relationships shape ecological properties of the human gut microbiome. *Nat Microbiol* **1**, 16088 (2016).
10. Valles-Colomer, M. *et al.* The neuroactive potential of the human gut microbiota in quality of life and depression. *Nat Microbiol* **4**, 623–632 (2019).
11. Zhou, H., He, K., Chen, J. & Zhang, X. LinDA: linear models for differential abundance analysis of microbiome compositional data. *Genome Biol* **23**, (2022).
12. Anderson, M. J., Ellingsen, K. E. & McArdle, B. H. Multivariate dispersion as a measure of beta diversity. *Ecol Lett* **9**, 683–693 (2006).
13. Office for National Statistics. Population estimates for the UK, England and Wales, Scotland and Northern Ireland. *Statistical bulletin* https://www.ons.gov.uk/peoplepopulationandcommunity/populationandmigration/populationestimates/bulletins/annualmidyearpopulationestimates/mid2017 (2017).
14. Kingston, A. *et al.* Is late-life dependency increasing or not? A comparison of the Cognitive Function and Ageing Studies (CFAS). *The Lancet* **390**, 1676–84 (2017).
15. Welch, A. A. Nutritional influences on age-related skeletal muscle loss. *Proceedings of the Nutrition Society* **73**, 16–33 (2014).

16. Rejc, E. *et al.* Loss of maximal explosive power of lower limbs after 2 weeks of disuse and incomplete recovery after retraining in older adults. *The Journal of Physiology* **596**, 647–665 (2018).

17. Houston, D. K. *et al.* Dietary protein intake is associated with lean mass change in older, community-dwelling adults: the Health , Aging, and Body Composition (Health ABC) Study. *Am J Clin Nutr 2008;87:150–5.* **87**, 150–155 (2008).

18. Ni Lochlainn, M., Bowyer, R. & Steves, C. Dietary Protein and Muscle in Aging People: The Potential Role of the Gut Microbiome. *Nutrients* **10**, 929 (2018).

19. Deutz, N. E. P. *et al.* Protein intake and exercise for optimal muscle function with aging: Recommendations from the ESPEN Expert Group. *Clin Nutr. 2014* **33**, 929–936 (2014).

20. Tieland, M., Borgonjen-Van Den Berg, K. J., Van Loon, L. J. C. & De Groot, L. C. P. G. M. Dietary protein intake in community-dwelling, frail, and institutionalized elderly people: Scope for improvement. *Eur J Nutr* **51**, 173–179 (2012).

21. Vigotsky, A. D., Schoenfeld, B. J., Than, C. & Brown, J. M. Methods matter: The relationship between strength and hypertrophy depends on methods of measurement and analysis. *PeerJ* (2018) doi:10.7717/peerj.5071.

22. Biagi, E. *et al.* Through Ageing, and Beyond: Gut Microbiota and Inflammatory Status in Seniors and Centenarians. *PLoS ONE* **5**, e10667 (2010).

23. Ridaura, V. K. *et al.* Gut microbiota from twins discordant for obesity modulate metabolism in mice. *Science* **341**, 1241214 (2013).

24. Smith, P. *et al.* Regulation of Life Span by the Gut Microbiota in The Short-Lived African Turquoise Killifish. *bioRxiv* 120980 (2017) doi:10.1101/120980.

25. Buigues, C. *et al.* Effect of a Prebiotic Formulation on Frailty Syndrome: A Randomized, Double-Blind Clinical Trial. *International journal of molecular sciences* **17**, (2016).

26. Theou, O. *et al.* Can a Prebiotic Formulation Reduce Frailty Levels in Older People? *The Journal of frailty & aging* **8**, 48–52 (2019).

27. Walker, S. N. *et al.* Maintenance of activity and eating change after a clinical trial of tailored newsletters with older rural women. *Nurs Res* **59**, 311–321 (2010).

28. Cesari, M. *et al.* Added Value of Physical Performance Measures in Predicting Adverse Health-Related Events: Results from the Health, Aging, and Body Composition Study. *J Am Geriatr Soc* **57**, 251–259 (2009).

29. Harvey, N. C. *et al.* Measures of Physical Performance and Muscle Strength as Predictors of Fracture Risk Independent of FRAX, Falls, and aBMD: A Meta-Analysis of the Osteoporotic Fractures in Men (MrOS) Study. *Journal of Bone and Mineral Research* **33**, 2150–2157 (2018).

30. RStudio Team. RStudio: Integrated Development for R. *RStudio, Inc., Boston, MA* (2015).

31. Welch, A. A. Nutritional influences on age-related skeletal muscle loss. *Proceedings of the Nutrition Society* **73**, 16–33 (2014).

32. Deutz, N. E. P. *et al.* Protein intake and exercise for optimal muscle function with aging: Recommendations from the ESPEN Expert Group. *Clin Nutr. 2014* **33**, 929–936 (2014).

33. Biagi, E. *et al.* Through Ageing, and Beyond: Gut Microbiota and Inflammatory Status in Seniors and Centenarians. *PLoS One* **5**, e10667 (2010).

34. Ridaura, V. K. *et al.* Gut microbiota from twins discordant for obesity modulate metabolism in mice. *Science (1979)* **341**, 1241214 (2013).

35. E9 Expert Working Group. ICH Harmonised Tripartite Guideline. Statistical principles for clinical trials. International Conference on Harmonisation. *Stat Med* **18**, 1905–42 (1999).

36. Ni Lochlainn, M. *et al.* The PROMOTe study: targeting the gut microbiome with prebiotics to overcome age-related anabolic resistance: protocol for a double-blinded, randomised, placebo-controlled trial. *BMC Geriatr* **21**, 407 (2021).

37. Bowyer, R. C. E. *et al.* Use of dietary indices to control for diet in human gut microbiota studies. *Microbiome* **6**, 77 (2018).

38. Searle, S. D., Mitnitski, A., Gahbauer, E. A., Gill, T. M. & Rockwood, K. A standard procedure for creating a frailty index. *BMC Geriatr* **8**, 24 (2008).

39. Cox, N. J. *et al.* The composition of the gut microbiome differs among community dwelling older people with good and poor appetite. *J Cachexia Sarcopenia Muscle* **12**, 368–377 (2021).

40. Wilson, M.-M. G. M. G. *et al.* Appetite assessment: simple appetite questionnaire predicts weight loss in community-dwelling adults and nursing home residents. *Am J Clin Nutr* **82**, 1074–1081 (2005).

41. Ipaq.ki.se. *Guidelines for Data Processing and Analysis of the International Physical Activity Questionnaire (IPAQ)-Short Form*. www.ipaq.ki.se. (2004).

42. CRAIG, C. L. *et al.* International Physical Activity Questionnaire: 12-Country Reliability and Validity. *Med Sci Sports Exerc* **35**, 1381–1395 (2003).

43. Cruz-Jentoft, A. J. *et al.* Sarcopenia: revised European consensus on definition and diagnosis. *Age Ageing* **48**, 16–31 (2019).

44. Black, A. E. The sensitivity and specificity of the Goldberg cut-off for EI:BMR for identifying diet reports of poor validity. *Eur J Clin Nutr* **54**, 395–404 (2000).

45. Black, A. E. Critical evaluation of energy intake using the Goldberg cut-off for energy intake:basal metabolic rate. A practical guide to its calculation, use and limitations. *Int J Obes* **24**, 1119–1130 (2000).

46. Roza, A. M. & Shizgal, H. M. The Harris Benedict equation reevaluated: resting energy requirements and the body cell mass. *Am J Clin Nutr* **40**, 168–182 (1984).

47. Dietary reference values for food energy and nutrients for the United Kingdom. Report of the Panel on Dietary Reference Values of the Committee on Medical Aspects of Food Policy - PubMed. https://pubmed.ncbi.nlm.nih.gov/1961974/.

48. RStudio Team. RStudio: Integrated Development for R. *RStudio, Inc., Boston, MA* (2015).

49. Kahan, B. C., Jairath, V., Doré, C. J. & Morris, T. P. The risks and rewards of covariate adjustment in randomized trials: an assessment of 12 outcomes from 8 studies. *Trials* **15**, 139 (2014).

50. White, I. R., Horton, N. J., Carpenter, J., Statistics, r. i. m. a. s. & Pocock, S. J. Strategy for intention to treat analysis in randomised trials with missing outcome data. *BMJ* **342**, d40–d40 (2011).

4 Study Protocol

THE PROMOTE STUDY

DIETARY PROTEIN AND SKELETAL MUSCLE IN OLDER TWINS: TARGETING THE GUT MICROBIOME TO OVERCOME ANABOLIC RESISTANCE

1. Protocol details

4.1 PROTOCOL TITLE:

Dietary Protein and Skeletal Muscle in Older Twins - Targeting the Gut Microbiome to Overcome Anabolic Resistance:

The PROMOTe Study

1.2 Names (titles), roles and contact details of:

Sponsor

King's College London

Professor Reza Razavi

Vice President & Vice Principal (Research)

Room 5.31, James Clerk Maxwell Building

57 Waterloo Road

London SE1 8WA

Tel: +44 (0)207 8483224

Email: reza.razavi@kcl.ac.uk

Co-Sponsor

Guy's and St Thomas' NHS Foundation Trust

Rachel Fay

R&D Office

16th Floor, Tower Block

Guy's Hospital

SE1 9RT

020 718 87188

Email: R&D@gstt.nhs.uk

Chief Investigator

Dr Claire Steves

Department for Twin Research

King's College London

3rd Floor South Wing, St Thomas' Hospital

SE1 7EH, London,

United Kingdom.

02071886765

Email: Claire.j.steves@kcl.ac.uk

Name and address of Investigator(s)

Name: Dr Mary Ni Lochlainn

Address: Department for Twin Research

King's College London

3rd Floor South Wing, St Thomas' Hospital

SE1 7EH, London

Telephone: 07784454066

Email: mary.ni_lochlainn@kcl.ac.uk

Name: Professor Kevin Whelan

Address: Department of Nutritional Sciences

King's College London

Room 4.68, Franklin Wilkins Building, 150 Stamford Street

SE1 9NH, London

Telephone: 02078484419

Email: kevin.whelan@kcl.ac.uk

5 Sponsor Statement

The Chief Investigator and the R&D (sponsor office) have discussed this protocol. The investigators agree to perform the investigations and to abide by this protocol

The investigator agrees to conduct the trial in compliance with the approved protocol, EU GCP, the UK Data Protection Act (1998), the Trust Information Governance Policy (or other local equivalent), the Research Governance Framework (2005' 2nd Edition; as amended), the Sponsor's SOPs, and other regulatory requirements as amended.

Contents Page

1. Protocol details 40
1.1 PROTOCOL TITLE: 40
2 Signature Page .... **Error! Bookmark not defined.**
Contents Page....................43
3 List of Abbreviations 44
4 Summary/Synopsis 45
5 Introduction 47
6 Trial objectives and purpose 48
7 Study design & Flowchart 49
7.1 Study Design 49
7.2 Flowchart 53
8 Study Population 54
8.1 Subject inclusion criteria 54
8.2 Subject exclusion criteria 55
9 Study procedures 55
9.1 Subject recruitment 55
9.2 Screening Procedures 56
9.3 Randomisation Procedures 56
9.4 Food Supplements 57
9.5 End of Study Definition 57
9.6 Follow up Procedures 57
10 Laboratories 57
10.1 Sample Collection/Labelling/Logging 57
10.2 Sample Analysis Procedures 57
10.3 Sample Storage Procedures 57
10.4 Data Recording/Reporting 58
10.5 Sample Transfer to sites outside the Organisation 59
11 Assessment of Safety 59
11.1 Definitions 59
11.2 Reporting Procedures: 60
11.2.1 Serious AEs: 60
11.2.3 Responsibilities 60
11.3 Ethics & Regulatory Approvals: 60
12 Compliance and withdrawal 61
12.1 Subject compliance 61
12.2 Withdrawal / dropout of subjects 61
13 Data 62
13.1 Data handling and record keeping 62
14 Statistical considerations 62
14.1 Sample size calculation 62
14.2 Statistical analysis 62
14.3 Interim analysis and data monitoring 63
14.3.1 Stopping / discontinuation rules and breaking of randomisation code 63
14.3.2 Monitoring, quality control and assurance 63
15 Financing and Insurance 64
16 Reporting and Dissemination 64
17 References 65

6 List of Abbreviations

AE	Adverse Event
ASV	Amplicon Sequence Variant
CI	Chief Investigator
CRN	Clinical Research Network
DTR	Department of Twin Research
GCP	Good Clinical Practice
GSTFT	Guy's and St Thomas' NHS Foundation Trust
IPAQ	International Physical Activity Questionnaire
KCL	King's College London
KCTU	King's College London Clinical Trials Unit
NHS	National Health Service
NIHR	National Institute of Health Research
NRES	National Research Ethics Service
OUT	Operational taxonomic unit
PI	Principal Investigator
QA	Quality Assurance
QC	Quality Control
R&D	Research & Development
REC	Research Ethics Committee
SAE	Serious Adverse Event
SOP	Standard Operating Procedure
SPPB	Short Physical Performance Battery

7 Summary/Synopsis

Title	Dietary Protein and Skeletal Muscle in Older Twins: Targeting the Gut Microbiome to Overcome Anabolic Resistance (The PROMOTe Study)
Protocol Short Title/Acronym	PROtein and Muscle in Older Twins: The PROMOTe Study.
Protocol Version number and Date	Version 1.0, 07/03/2021
Is the study a Pilot?	No
IRAS Number	257415
REC Reference	19/NW/0187
Sponsor Reference	257415
Study Duration	24 Months
Methodology	Randomised controlled, double blinded, dietary intervention study
Sponsor name	King's College London
Co-Sponsor name	Guy's and St Thomas' NHS Trust
Chief Investigator	Dr Claire Steves
Funder Name	National Institutes of Health Research (NIHR)
Medical condition or disease under investigation	General health; ageing; muscle ageing
Purpose of clinical trial	A study to assess the effect of prebiotic food supplementation in combination with protein supplementation on muscle strength in older twins. This will provide evidence for the role of the gut microbiome in anabolic resistance to dietary protein. This study will also test whether these supplements result in measurable improvements in other markers of muscle health and physical activity, markers of gut microbiome health, metabolites, anthropological measurements, and cognition.
Primary objective	Test whether modulation of the gut microbiome using a prebiotic food supplement, in addition to protein supplementation, can improve muscle strength (as measured by chair-rise time) versus protein supplementation alone
Secondary objective (s)	Test whether modulation of the gut microbiome using a prebiotic food supplement, in addition to protein supplementation, has an effect on short physical performance battery, gut microbiome composition, serum metabolites, cognition, and a range of other phenotypical markers, versus protein supplementation alone.

Number of Subjects/Patients	The sample size has been calculated to detect effect in chair-rise time; 70 individuals will have 80% power at 0.05 alpha to identify a difference of 3.5 seconds (effect size in 35 people per group, over 12 weeks) (20% dropout rate).
Trial Design	Randomised controlled double blinded dietary intervention study
Main Inclusion Criteria	Participant eligibility includes those aged >60 years who have a dietary protein intake below 1.3g/kg/day.<br><br>Participants must be able to consent.<br><br>Participants must have access to a computer/tablet or other similar device.
Statistical Methodology and Analysis	Analysis will assess microbiota differences between the two study arms and between twin pairs. Microbiome composition will be analysed with respect to (1) species diversity, (2) compositional differences, and (3) differences in abundance of taxa.<br><br>All twins within each study arm will be used in paired tests to assess if microbiota dissimilarity increased with differences in muscle strength, taking relevant covariates into consideration.<br><br>Secondary analyses will include linear mixed effects regression models to investigate associations between microbiota composition, microbial metabolite markers, markers of appetite and other lifestyle and physiological parameters including muscle strength (as measured by chair-rise time) adjusting for covariates and multiple testing.

8 Introduction

The age of populations worldwide is increasing. Nearly 12 million UK residents were aged >=65 years in mid-2017; 18.2% of the population [13]. With this ageing population comes an increase in age-related conditions and time spent living with age-related morbidity [14].

Skeletal muscle has several important functions beyond locomotion, including protein metabolism [15]. Age-associated muscle loss is gradual; typically there's a greater loss of type II fibres; the main ones involved in preventing a fall [15]. Older people lose more muscle with bedrest and show an attenuated response to retraining after immobilisation, in comparison to younger individuals [16].

In the Health ABC Study, older participants in the highest protein intake quintile lost ~40% less appendicular lean mass than those in the lowest quintile, over three years [17]. High protein intake is associated with reduced rehabilitation time, better cardiovascular function, and improved mortality [18]. Unfortunately, several factors can lead to reduced protein intake in older age, including social isolation, dysphagia, slower gastric emptying etc.

In addition to taking less protein in their diet, research has shown that older adults display anabolic resistance to protein intake, a blunted responsiveness of older muscle in terms of muscle protein synthesis, compared with younger adults [15]. This has led to a higher daily intake of 1-1.3g/kg/day being recommended by experts [19]. Many older people do not meet this [20], and this was reflected within our TwinsUK dataset; 30% of those over 60 years were below 1g/kg/day. The current UK Reference Nutrient Intake for adults is 0.8 g/kg/day.

Skeletal muscle mass is regulated by the processes of muscle protein synthesis and breakdown (MPS and MPB). MPS rates are largely controlled by responsiveness to anabolic stimuli, e.g. consumption of food, and physical activity. Catabolic stressors include illness, physical inactivity and inflammation, of which older people tend to have higher rates. The aetiology of anabolic resistance is complex, involving aging physiology and physical inactivity. The multiple mechanisms postulated involve impairments at most levels of protein metabolism. Muscle mass typically corresponds with muscle strength [21], however this has not been vigorously tested in older adults and depends greatly on which measurements are used.

The gut microbiome is composed of bacteria, archaea, viruses, and eukaryotic microbes that reside in the gut. Its role in maintaining healthy physiology is a rapidly evolving field of enquiry. With age, the resilience of the gut microbiome is reduced, as it becomes more vulnerable to lifestyle changes, with changed species richness and increased inter-individual variability [22].

The potential of the gut microbiota to alter physiology has been shown by landmark animal faecal transplant studies, which have demonstrated body composition changes in the recipient reflective of the donor's phenotype [23]. This highlights the role of microbiota in characterising metabolic phenotypes. Amazingly, a recent study showed that transferring gut microbes of young killifish to

older ones ameliorated ageing conditions, extending the lifespan of the older fish [24]. Notably, the transplanted older fish also displayed increased 'spontaneous exploratory behaviour', essentially physical activity.

Several different mechanisms have been proposed for anabolic resistance, and the gut microbiome plays a role in many of these. Examples include protein digestion and absorption, gut barrier function, and inflammation [18]. This study aims to assess whether the gut microbiome modulates anabolic resistance, therefore presenting a malleable therapeutic target for prevention and reversal of muscle loss with age. Current therapies are limited to dietary recommendations, nutritional supplements and exercise programmes. For many older adults a rigorous exercise programme is simply not feasible, be it due to medical reasons, personal choice or a lack of resources.

We have selected an older study population who have a lower than recommended protein intake, and who are weaker, with below average diversity of their gut microbiome. This population represents those who stand to benefit most from a dietary intervention strategy such as the protein plus prebiotic food supplement we are testing in this trial.

In light of the COVID-19 global pandemic and the resulting restrictions on travel, this protocol has been adapted such that all 'visits' will be carried out online, via video teleconferencing software. Participants will take part from home, with the study visits taking place over video teleconferencing. The landscape of research will change drastically in the post COVID-19 era and research carried out remotely, utilising technology and innovative techniques, will become both more common and necessary. A subset of the study population will be invited to the department for repeat physical measures for validation purposes, which will be optional to them.

9 Trial objectives and purpose

The current study's objective is to test whether prebiotic food supplementation in combination with protein supplementation improves muscle strength in older ambulant community dwelling individuals.

Specific Aim 1: carry out an interventional dietary study to test whether modulation of the gut microbiome, in addition to protein supplementation, can improve skeletal muscle function (muscle strength) versus protein supplementation alone.

Specific Aim 2: To deliver a wealth of data, including effect sizes, on specific causal links between protein supplementation, prebiotic food supplementation, changes in the gut microbiome composition, changes in the metabolomic profile and changes in skeletal muscle function of an individual.

10 Study design & Flowchart

10.1 Study Design

The proposed study will provide a simple but robust intervention study using protein supplementation and prebiotic food supplementation to improve our understanding of the mechanisms linking nutrition to health outcomes that are mediated by the gut microbiome.

The study will be a randomised, placebo controlled, double blinded, intervention study in which participants will be grouped into two different arms. Randomisation will control for equal distribution of key characteristics that may confound between group comparisons and will be assessed by analysis of pre-existing baseline data. Participants will be randomised as twin pairs, with one twin from the pair in the prebiotic food supplement arm and one twin from the pair in the placebo arm. Twin pairs are matched for age and often for sex, early environment, baseline genetic sequence, socioeconomic status etc., proving a strong model for detecting changes that are truly significant.

Stage 1: Participant recruitment

Participants will be selected in twin pairs, from the TwinsUK registry on the basis of the following characteristics:

1. Dietary protein intake below optimal for older adults (1-1.3g/kg/day)
   (in order to target those who are most likely to see clinical benefit). We will use existing protein intake data to identify these individuals.
2. Aged ≥60 years (representing the typical population affected by muscle ageing)
   (We have 1314 registered volunteers >60 years with protein intake <1.3g/kg/day from which to recruit)

Studies within TwinsUK have an excellent record in recruitment. Volunteers who are below the mean in chair-rise time and shannon diversity will be preferentially recruited first, with recruitment extending to those above the mean if necessary (increasing ability to detect health effect). Shannon diversity is a marker of the diversity of the gut microbiome; a more diverse microbiome is typically considered healthier. We hypothesise that individuals with a lower than average Shannon diversity will show a greater response to the prebiotic food supplement intervention.

Once identified as eligible using pre-existing data, participants will be invited to take part. The study will be explained on the phone, eligibility will be assessed, and online consent taken (see Section 9.1). Once consent is completed, they will be invited for a baseline virtual visit. They will be sent questionnaires covering appetite, diet, quality of life, and physical activity, and will be asked to keep a 3-day food diary prior to their first virtual visit. Participants will also be provided with a link to carry out an online cognitive assessment (CANTAB). A postal pack will be sent out containing all the necessary items to collect samples and carry out all measures during the virtual visits.

After the baseline visit, participants will undergo computer generated randomisation, as twin pairs, completed by the King's College London Clinical Trials Unit (see Section 9.3). Participants will be allocated into two arms, either the protein supplement plus placebo (maltrodextrin) intake arm or

the protein supplement plus prebiotic food supplement intake arm. After randomisation the supplements will be posted to the participant. Researchers and participants will be blinded as to which group each participant is in. The Kings Clinical Trials Unit will liaise directly with the company providing the supplements to ensure complete blinding of research team. A designated member of the departmental administration team will have access to the unblinded information.

Stage 2: Baseline measures (visit 1):

Sampling:

Stool and urine samples will be collected by the participants themselves using the sample collection kits that will be provided to them in the postal pack. Capillary blood, and saliva samples will also be collected by the participant. An explanatory pack will be posted with the necessary apparatus to take the sample. Participants can carry this out under guidance from the researcher on the video teleconference. Return envelopes will be provided for all samples to be posted back to the department.

Examination:

Measurements will be taken by the participant with a measuring tape provided in the postal pack. Participants will be asked to weight themselves if they have a weighing scales. Weighing scales can be calibrated using a standard household item, such as a tin of beans. Baseline Short Physical Performance Battery (SPPB) will be carried out remotely (this includes chair-rise time), with live instructions from a trained researcher. A dynamometer will be provided to all participants via post in order to measure handgrip strength, and this measurement will be taken with guidance from the trained researcher, during the video call. Any queries regarding the questionnaire, food diary, or cognitive assessment can be addressed.

Stage 3: Dietary intervention

Control and treatment arms: All participants will be provided with a sachet of powdered food supplement to be taken daily with a glass of water or other drink (hot or cold). All sachets will contain leucine-rich protein supplement.

Sachets for participants in the placebo arm will also contain 20 g of maltrodextrin powder (placebo). Sachets for participants in the intervention arm will contain prebiotic food supplement. All participants will be asked to take 1 sachet daily, for a period of 12 weeks. These supplement sachets in each arm will be indistinguishable.

Regular contact will be made with participants by phone/email to encourage compliance and assess for any adverse effects. All participants will be encouraged to engage in regular resistance exercise at least twice per week throughout the intervention and will be provided with written advice on this at the beginning of the study.

Stage 4: Follow up/end of study measures (visit 2)

At the end of 12 weeks, participants will be asked to collect stool and urine samples again using the sample collection kits that will be provided to them in their postal pack. Capillary blood, and saliva samples will be collected by the participant, with guidance from a trained researcher, while on the video call. Measurements, and SPPB assessment will be repeated. In advance of the final visit, participants will be asked to complete another 3-day food diary, cognitive test, and to fill in another questionnaire, both of which will be provided in the postal pack, or online.

If participants need more flexibility on the date of their final visit, more sachets can be posted to them to continue the intervention until their final virtual visit, to a maximum of one month's additional intervention time.

Primary outcome: chair rise time

Secondary outcomes:

Grip strength (kg)

Short physical performance battery

IPAQ score - physical activity questionnaire

Gut microbiome measures

Serum metabolites

Frailty Index

Salivary microbiome measures

Urinary microbiome measures

Changes in cognition (as measured by CANTAB battery)

Changes in appetite (as measures by SNAQ questionnaire)

Stage 5: Validation measures for subset of study population

COVID-19 pandemic rules allowing, a subset of the study population will be invited to take part in a visit in-person to the department for repeat measures of those taken in the virtual visits. This will include physical measures, anthropological measures, and biological samples. Questionnaires will not be repeated. Participants will be invited at random, as twin pairs. The visit will be arranged to take place close to the final virtual visit, in order to provide accurate validation measures for those measures taken remotely. This part of the study may not be feasible if travel restrictions and lockdown measures remain in place, and will only be done if safe to do so. Those selected at random will be contacted by the research team to arrange an appointment. Taking part in this visit is optional for participants.

Stage 6: Data analysis

Microbiome analysis: stool samples collected and frozen within two hours will be assessed for markers of microbiome function and gut health. Blood samples will be collected for the analysis of metabolites. The vial will be frozen and stored. When all vials are collecting from every participant these will be sent for external processing of the metabolite profile of the serum. Urine and salivary samples will be collected at the visits. These will undergo processing to assess the microbiota present in each sample. Statistical analysis will involve repeated measures mixed modelling of the change in outcome measures between interventions.

10.2 Flowchart

**Stage**	**1**	**2**	**3**	**4**	**5**	**6**	**7**
**Assessment**	**Eligibility**	**Pre-baseline video call**	**Baseline video call (week 1)**	**Week 3-5**	**Week 7-9**	**Pre-final call**	**Final video call (week 12)**
Inclusion/Exclusion Criteria checks	X						
Participant information and informed consent	X		X				
Randomisation			X				
Postal box sent out		X					
3-day food diary (post/online)		X				X	
Questionnaire (post/online)		X				X	
CANTAB cognitive test (online)		X				X	
Provide supplements (post)			X				
Faecal sample (post)			X			X	
Capillary blood sample (post)			X				X
Urine sample (post)			X			X	
Saliva sample (post)			X				X
Short Physical Performance Battery			X				X
Weight (kg) – if scales available			X				X
Height (cm)			X				X
Telephone calls to check compliance/adverse effects				X	X		

Participants to count leftover supplement sachets							X

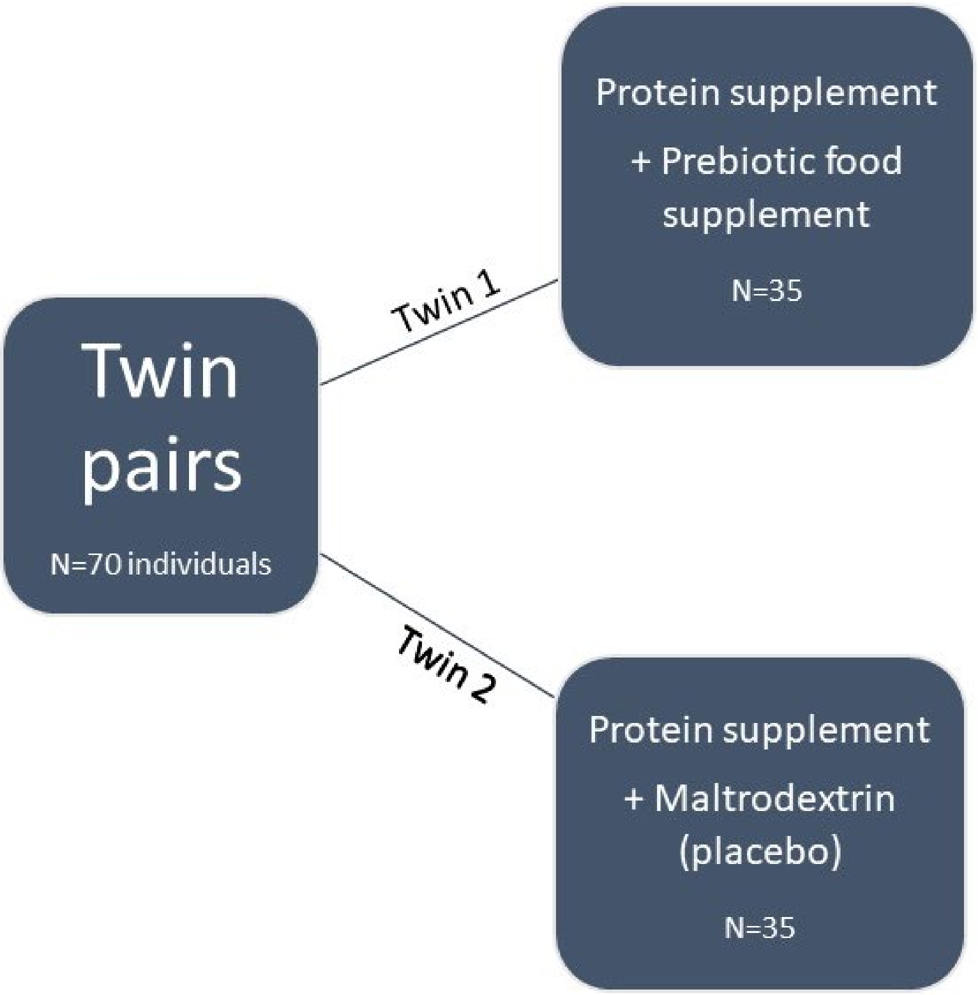

11 Study Population

Participants will be recruited from the TwinsUK databases and invited to attend a virtual visit using video teleconferencing. A subset will later be invited to the department in St Thomas' Hospital to repeat the physical performance measures.

11.1 Subject inclusion criteria

-Aged >=60 years

-Dietary protein intake of <1.3g/kg/day

-Able to consent

-Must have access to video teleconferencing on a computer, laptop, tablet or phone device

11.2 Subject exclusion criteria

-Severe food allergy

-Current or recent antibiotic use (preceding 3 months)

-Currently or recent use of protein or leucine supplements (preceding 3 months)

-Currently or recent use of probiotic or prebiotic food supplements (preceding 3 months)

-Current or prior history of gastrointestinal disease e.g., gastrointestinal cancer, inflammatory bowel disease, bariatric surgery, irritable bowel syndrome

-Chronic Kidney Disease Stage 3 or higher (eGFR <=30mls/min)

-History of any significant injury or surgery which currently affects physical functioning and ability to undertake chair stand test

-Weight loss of ≥5% of body weight in preceding 6-12 months

-Currently involved in other intervention studies

-Any condition or circumstance likely to interfere with the normal conduct of the study and interpretation of the results, as judged by the investigators

As the study population are over 60 years old, it is assumed that there will be no pregnant women who are eligible to take part.

12 Study procedures

12.1 Subject recruitment

Participants will be approached either by the administrative team via telephone, email or letter and selection will be based on the inclusion and exclusion criteria outlined for the study.

The consenting process:

Potential volunteers will receive a phone call detailing what the study involves. Once they have stated their interest to participate, the administrative assistant will send the volunteer information sheet and consent form to the individual either by post or email. After reading the information sheet they can ask questions by ringing in or emailing, decided whether to continue.

If they are keen to proceed and take part in the study, they will be sent a link for an online consent form for them to sign. Twins who have consented will be enrolled into the study by a trained

member of the administration and data management team. They will then book an appointment for a virtual visit, and send out the postal pack.

Participants will not be paid for taking part in the study; however, all travel and hotel accommodation expenses will be paid for, for any participants who attend the department for validation measures.

12.2 Screening Procedures

Screening Assessment: Prospective participants will undergo a thorough screening questionnaire administered over the phone by a trained researcher. Once considered eligible, participants will be booked in for their baseline virtual visit. All patients that undergo screening are logged into a screening log associated with the study by a trained researcher part of the data management team at the Department of Twin Research, KCL.

12.3 Randomisation Procedures

Participants will undergo computer generated block randomisation in twin pairs by the King's College London Clinical Trials Unit (KCTU). A web-based randomisation system will be designed, using the bespoke KCTU randomisation system. The randomisation system will be created in collaboration with the trial analyst/s and the CI and maintained by the King's Clinical Trials Unit for the duration of the project. It will be hosted on a dedicated server within KCL.

The CI or delegate will request usernames and passwords from the KCTU. System access will be strictly restricted through user-specific passwords to the authorised research team members. It is a legal requirement that passwords to the randomisation system are not shared, and that only those authorised to access the system are allowed to do so. If new staff members join the study, a user-specific username and password must be requested via the CI or delegate (e.g. Trial Manager) from the KCTU team and a request for access to be revoked must be requested when staff members leave the project. Study site staff experiencing issues with system access or functionality should contact the CI or delegate (e.g. Trial Manager) in the first instance.

Participant initials and date of birth will be entered on the randomisation system, NHS number, email addresses, participant names and addresses and full postcodes will not be entered into the randomisation system. No data will be entered onto the randomisation system unless a participant has signed a consent form to participate in the trial. Randomisation will be undertaken by authorised staff onto the randomisation system by going to www.ctu.co.uk and clicking the link to access the randomisation system. A full audit trail of data entry will be automatically date and time stamped, alongside information about the user making the entry within the system.

The CI team will undertake appropriate reviews of the entered data, the purpose of data cleaning. No data can be amended in the system, however CI or delegate (e.g. Trial Manager) may request King's Clinical Trials Unit to add notes against individual subject entries to clarify data entry errors. Upon request, KCTU will provide a copy of the final exported dataset to the CI in .csv format and the CI will onward distribute as appropriate. Researchers and participants will be blinded as to which arm each participant is in.

12.4 Food Supplements

Commercially available protein supplements, prebiotic food supplements and maltrodextrin (placebo) will be purchased. Indistinguishable sachets will be made up by the company providing the prebiotic food supplement. These can be stored at room temperature. The prebiotic food supplement is called Darmocare Pre®. The composition is Inulin min. 3375mg, fructooligosaccharides (FOS) min. 3488mh per level measuring spoon. Excipients: guaranteed to contain no genetically modified organisms, maize, soy, yeast, gluten, lactose, added saccharose, gelatine, animal substances, preservatives, artificial coloring, flavoring and aromatic substances. Darmocare Pre® has previously been used in studies in older adults, with reported benefits on grip strength [25] and frailty index [26] versus placebo.

Participants can continue their normal diet otherwise. Participants will take their supplement daily mixed in a glass of water or other drink, at the same time of day each day for a 12-week period. All participants will be asked to bring back any remaining sachets at the end of the trial period to help us ascertain compliance.

12.5 End of Study Definition

Data collection complete and database lock.

12.6 Follow up Procedures

No follow up procedures will be required for this study as the participants included in the study are healthy volunteers.

13 Laboratories

13.1 Sample Collection/Labelling/Logging

All sample kits provided, and participant data will be pseudo-anonymised with a unique identifier, the samples may be barcoded to link with participants unique ID. The principal investigator will ensure that all data collected in the study, are recorded in a timely manner according to any instructions provided and the subject numbering process will commence at the point of informed consent. All sample collection kits provided to the participants will contain detailed instruction on storage conditions to ensure the integrity and viability of samples. Participants will be asked to record the date and time of collection for each sample they collect. The samples sent back to the laboratory will be logged in using the unique barcode and/or unique ID. A complete chain-of-custody will be maintained for all samples throughout, from point of acquisition, storage and all uses, including disposal where relevant.

13.2 Sample Analysis Procedures

Serum will be generated from the whole blood for metabolomics profiling.

Microbiome analysis: Faecal samples will be collected using a sample collection kit provided to the participants. 16s analysis sequencing, and shotgun metagenomics where possible, will be carried out at a reputable institution with a track record of such analyses as a service.

Microbiome markers: Stool samples collected and frozen will be assessed in greater detail for markers of microbiome function. Any remaining sample will be stored at the DTR for potential use in future analysis, such as to allow bridging to other new or improved technologies. The biological (faecal, urine, blood and saliva) samples will be stored at -80C upon receipt and subsequently used for microbiome and metabolomics analysis. These assays will be undertaken by the study investigators or designated collaborators either within or outside of the UK, provided the appropriate Material Transfer Agreements are in place.

13.3 Sample Storage Procedures

All samples collected will be stored at the Department of Twin Research laboratory at King's College London. Faecal, blood, saliva and urine samples will be stored at -80oC in separate locked, alarmed freezers with CO2 back up and 24/7 on call support. Once all collected, blood and stool samples will be sent to Metabolon, an external laboratory which will carry out the metabolite testing. This is the same lab the department has used for such testing in the past.

Biological samples for future research will be retained at by King's College London for an indefinite period. However, should the tissue have no obvious further use it will be destroyed.

The HTA licence details are as follows:

- Licensing Number: 12522
- Licensed Premises: King's College London, St Thomas' Campus, Westminster Bridge Road, London, SE1 7EH
- Licence Holder: King's College London
- Designated Individual: Fiona Sutherland

13.4 Data Recording/Reporting

Data entered into the database and on which the analysis will be performed are pseudonymised, using a unique identification number. At the point of publication, the identification numbers are not published.

A web based electronic data capture (EDC) system will be designed, using the InferMed Macro 4 system. The EDC will be created in collaboration with the trial analysts and the CI and maintained by the King's Clinical Trials Unit for the duration of the project. It will be hosted on a dedicated server within KCL.

The CI or delegate will request usernames and passwords from the KCTU. Database access will be strictly restricted through user-specific passwords to the authorised research team members. It is a legal requirement that passwords to the EDC are not shared, and that only those authorised to access the system are allowed to do so. If new staff members join the study, a user-specific username and password must be requested via the CI or delegate (e.g. Trial Manager) from the KCTU team and a request for access to be revoked must be requested when staff members leave the project. Study site

staff experiencing issues with system access or functionality should contact the CI or delegate (e.g. Trial Manager) in the first instance.

Participant initials and date of birth will be entered on the EDC, NHS number, email addressed, participant names and addresses, and full postcodes will not be entered into the EDC. No data will be entered onto the EDC system unless a participant has signed a consent form to participate in the trial. Source data will be entered by recruiting site staff, typically within 5 days of data collection by authorised staff onto the EDC by going to www.ctu.co.uk and clicking the link to access the MACRO 4 EDC system. A full audit trail of data entry and any subsequent changes to entered data will be automatically date and time stamped, alongside information about the user making the entry/changes within the system. The CI team will undertake appropriate reviews of the entered data, for the purpose of data cleaning and will request amendments as required.

At the end of the trial, the site PI will review all the data for each participant to verify that all the data are complete and correct. At this point, all data can be formally locked for analysis. Upon request, KCTU will provide a copy of the final exported dataset to the CI in .csv format and the CI will onward distribute as appropriate.

13.5 Sample Transfer to sites outside the Organisation

Samples may be shared with third parties (e.g. other laboratories) following agreement with King's College London which will comprise the Chief Investigator, the Principal Investigators, GSTT senior staff, professional researchers and lay persons. Transfer of tissue and data to a third party is subject to a separate Material Transfer Agreement issued by King's College London that holds the custodianship of those samples and data. Any tissue transferred outside King's College London will be used up, returned or destroyed once the specific research is completed.

14 Assessment of Safety

As this study is an intervention study, safety monitoring will focus on unanticipated events involving risks to participants, including unanticipated problems that meet the definition of a serious adverse event.

14.1 Definitions

Adverse event (AE): any untoward medical occurrence in a patient or study subject reported to the study team during in person and telephone visits.

Serious adverse event (SAE): any untoward and unexpected medical occurrence or event that;

1. Result in death
2. Is life threatening
3. Requires hospitalisation or prolongation of existing in-patient hospitalisation
4. Results in persistent or significant disability or incapacity

Medical judgement will be exercised in deciding whether an AE is serious. Important AEs that are not immediately life-threatening or do not result in death or hospitalisation but may jeopardise the subject or may require intervention to prevent one of the other outcomes listed in the definition above will also be considered serious.

14.2 Reporting Procedures:

Adverse events will be recorded and reported to the Chief Investigator. The event will also be documented and discussed with all members of the research group in the research departmental meetings.

11.2.1 Serious AEs:

All SAEs (both related and unrelated) will be recorded and reported immediately to the chief investigator and the sponsor. Relapse and death due to an unrelated or pre-existing condition, and hospitalisation for treatment of a pre-existing condition do not require reporting as SAEs.

All SAEs should be reported to the research ethics committee (REC) within 15 days of the chief investigator becoming aware where in the opinion of the Chief Investigator, the event was:

Related i.e. resulted from administration of any of the research procedures

Unexpected i.e. an event that is not listed in the protocol as an expected occurrence.

The SAE report form for non-CTIMPs will be used (available from NRES website) and will be sent to the main REC for the trial.

11.2.3 Responsibilities

The co-sponsors (King's College London and GSTFT) has delegated responsibility for the study to the chief investigator. The principal investigator at each site is responsible for reviewing all AEs and recording them appropriately in the study database.

14.3 Ethics & Regulatory Approvals:

This interventional study will be conducted in compliance with the principles of the Declaration of Helsinki (1996), the principles of GCP and in accordance with all applicable regulatory requirements including (but not limited to) the Research Governance Framework. The study may be subject to inspection and audit by King's College London and Guy's and St Thomas' NHS Foundation Trust under their remit as sponsors and by other regulatory bodies. This is to ensure adherence to GCP and the NHS Research Governance Framework for Health and Social Care (2nd edition).

This protocol and all related documents will be submitted for review to a Research Ethics Committee (REC). Any amendments to approved documents or newly created documents will likewise be submitted for approval. The study will be submitted for consideration for adoption on to the National Institutes for Health Research (NIHR) Clinical Research Network portfolio.

This study has been presented to the Department of Twin Research Volunteer Advisory Panel (VAP), who were in favour of it, and the documents have been reviewed by the eVAP, another group of volunteers who review materials remotely. The eVAP were also in favour of the study.

15 Compliance and withdrawal

15.1 Subject compliance

Regular monitoring of compliance will be achieved by regular contact with the participants either via email, text messaging, or telephone. Compliance will be judged by completion of the end of study questionnaires and by the remaining number of sachets at the end of the study. Twin pairs will be advised not to share each other's supplements.

15.2 Withdrawal / dropout of subjects

Participants can withdraw their consent, without giving any reason at any time during the study by contacting the study contacts. Similarly, the participant can withdraw their consent for the continued retention and use of their samples even after they have been collected, without giving a reason. Reason for discontinuation from the study will be asked about sensitively and recorded. If a subject withdraws consent to the use of donated biological samples, the retained samples will be disposed of/destroyed.

If a participant, who has given informed consent, loses capacity to consent during the study, the participant would be withdrawn from the study. Identifiable data or tissue already collected with consent would be retained and used in the study. No further data or tissue would be collected, or any other research procedures carried out on or in relation to the participant.

The Chief Investigator:

- Will ensure subjects' withdrawal of informed consent is notified immediately to Guy's and St Thomas' NHS Foundation Trust and King's College London.
- Will ensure that biological samples from that subject, if stored at the study site, are immediately identified, disposed of/destroyed and the action documented.
- Will ensure that the laboratory(ies) holding the samples is/are informed about the withdrawn consent immediately and that samples are disposed of/destroyed and the action documented and returned to the study site.

Guy's and St Thomas' NHS Foundation Trust and the King's College London will ensure that the central laboratory(ies) holding the samples is/are informed about the withdrawn consent immediately and that samples are disposed of/destroyed and the action documented returned to the study site. In the event that analysis/research has already been performed, Guy's and St Thomas' NHS Foundation Trust and King's College London will retain the results and associated data for regulatory reasons but individual data that can be identified as originating from that subject will not be used in any subsequent analyses.

16 Data

16.1 Data handling and record keeping

The Chief Investigator will act as custodian for the study data. The following guidelines will be strictly adhered to:

(1) Completed paper case report forms should be stored within the study site file.

IRAS project ID: 257415 Version 1.0

(2) The study site file will be located in a locked storage with access restricted to permitted research staff.

(3) All study data will be stored and archived in line with the Medicines for Human Use (Clinical Trials) Amended Regulations 2006.

17 Statistical considerations

17.1 Sample size calculation

From our existing data, we have observed that chair-rise time is to a good approximation log normal, with log10(time) having a SD of 0.126. We consider a relative reduction in average time of 20% to be both clinically important and plausible. Based on these figures, we would need complete data on 30 subjects per group (60 in total) for 80% power. Allowing for 20% dropouts, we would need 70 (35 per group) recruited. Based on the numbers we plan to recruit for the study, we estimate a recruitment time period of 18 months.

We have estimated a powered sample size based on other studies using chair-rise time [27–29], however we note that no study has looked at this in the context of protein and/or prebiotic food supplementation and therefore may not be accurate.

17.2 Statistical analysis

Analysis will assess microbiota differences between the two study arms and between twin pairs. Microbiome composition will be analysed with respect to (1) species diversity, (2) compositional differences, and (3) differences in abundance of taxa. Analysis will be carried out using statistical programs STATA and RStudio [30]. All analysis will take a hierarchical approach; crude models assessing just the variable of interest (prebiotic supplementation), and models adjusted for key biological (age, gender, exercise etc..) and technical covariates (e.g. differences in library size) will be applied.

Differences in species diversity and richness will be compared between each study arm in regression analysis in crude and adjusted models. Distance matrixes characterise the inter-individual differences in microbiota composition; ordination analyses and permutational multivariate analysis of variance (PERMANOVA) will be used to understand differences in composition between each study arm.

ASVs will be collapsed to family, order, and phylum levels, with hierarchical models, adjusted for each potential mediator individually, then fully adjusted. All models will be adjusted for multiple testing.

All twins within each study arm will be used in paired tests to assess if microbiota dissimilarity increased with differences in muscle strength, taking relevant covariates into consideration. An intention to treat analysis will be carried out to address participants who do not complete the study.

Secondary analyses will include linear mixed effects regression models to investigate associations between microbiota composition, microbial metabolite markers, markers of appetite and other lifestyle and physiological parameters including muscle strength (as measured by chair-rise time) adjusting for covariates and multiple testing.

17.3 Interim analysis and data monitoring

17.3.1 Stopping / discontinuation rules and breaking of randomisation code

It is not envisaged that circumstances will arise that require termination of the trial given the dietary nature of the study. The trial may be prematurely discontinued by the sponsor, CI or regulatory authority on the basis of new safety information or for any other reason given by the ethics committee concerned.

17.3.2 Monitoring, quality control and assurance

Data custodians ensure that important datasets are received from external sources, maintained and are accessible by the appropriate users. The IT and Data Manager are in charge of overseeing all the aspects of data management and help to ensure that datasets

maintain their integrity and do not become compromised. IT support ensures the maintenance and security of hardware, software and network equipment used to access the data whilst the Database Administrator ensures the safety, integrity and security of the data and database management software.

The Database Developer creates applications that help extract data, provide metadata and generally turn datasets into useful information. They also provide the framework for the security of our data and an infrastructure to realise our Open Access ethos.

Principles of data quality are applied at all stages of the data management process (capture, digitisation, storage, analysis, presentation and usage). The DTR data team carries out both quality assurance (QA) and quality control (QC) on a continuous basis, with periodic audits, cleaning, harmonising and removing duplicate records. A single view of the data is created if it is stored in multiple disparate systems. We endeavour to reach 85%-90% accuracy considering two factors:

frequency of incorrect data fields or records and significance of the data field with the error. We use standardised survey methodologies wherever possible to reduce errors.

As part of the QC process, data quality is assessed by applying verification and validation procedures. Our data entry officers (usually outsourced to professional data entry companies) perform data verification checks and make sure that the digitised data entered matches the source data. Data are often automatically scanned but verified by a human being. Double blind entry is also used. Expert staff perform data validation checks to check, for example, the units and ranges provided by the Principal Investigator (PI). This step can also contain manual and automated algorithmic batch checks. To reduce errors of omission, at the beginning of a project PIs are required to describe all data and the equipment that collects the data including units and ranges.

Before any data are allowed to be imported in the Phenobase we require clear documentation to be provided and stored with the data. This requires (but is not limited to), 1) metadata that records information at the dataset level describing what the data represent and, 2) what data checks have been done and what changes have been made to each record.

18 Financing and Insurance

This study is funded by the National Institute of Health Research (NIHR). Participants will not be paid to take part in the study. However, all travel expenses to and from St Thomas' Hospital will be covered.

This study is co-sponsored by King's College London and Guy's and St Thomas' NHS Foundation Trust. The sponsors will at all times maintain adequate insurance in relation to the study independently. King's College London, through its own professional indemnity (Clinical Trials) and no fault compensation and the Trust's duty of care to patients via NHS indemnity cover, in respect of any claims arising as a result of clinical negligence by its employees, brought by or on behalf of a study patient.

19 Reporting and Dissemination

The results of the study will be reported and disseminated at international conferences and in peer-reviewed journals, but the participant's identity will not be revealed. The chief investigator and co-investigators will ensure that on completion of the study, the results are analysed, documented and reported.

20 References

1. Pasolli, E. *et al.* Extensive Unexplored Human Microbiome Diversity Revealed by Over 150,000 Genomes from Metagenomes Spanning Age, Geography, and Lifestyle. *Cell* **176**, 649-662.e20 (2019).

2. Nielsen, H. B. *et al.* Identification and assembly of genomes and genetic elements in complex metagenomic samples without using reference genomes. *Nat Biotechnol* **32**, 822–828 (2014).

3. Parks, D. H., Imelfort, M., Skennerton, C. T., Hugenholtz, P. & Tyson, G. W. CheckM: assessing the quality of microbial genomes recovered from isolates, single cells, and metagenomes. *Genome Res* **25**, 1043–1055 (2015).

4. Langmead, B. & Salzberg, S. L. Fast gapped-read alignment with Bowtie 2. *Nat Methods* **9**, 357–359 (2012).

5. Schubert, M., Lindgreen, S. & Orlando, L. AdapterRemoval v2: rapid adapter trimming, identification, and read merging. *BMC Res Notes* **9**, 88 (2016).

6. Li, H. & Durbin, R. Fast and accurate short read alignment with Burrows–Wheeler transform. *Bioinformatics* **25**, 1754–1760 (2009).

7. Huerta-Cepas, J. *et al.* Fast Genome-Wide Functional Annotation through Orthology Assignment by eggNOG-Mapper. *Mol Biol Evol* **34**, 2115–2122 (2017).

8. Kanehisa, M. KEGG: Kyoto Encyclopedia of Genes and Genomes. *Nucleic Acids Res* **28**, 27–30 (2000).

9. Vieira-Silva, S. *et al.* Species–function relationships shape ecological properties of the human gut microbiome. *Nat Microbiol* **1**, 16088 (2016).

10. Valles-Colomer, M. *et al.* The neuroactive potential of the human gut microbiota in quality of life and depression. *Nat Microbiol* **4**, 623–632 (2019).

11. Zhou, H., He, K., Chen, J. & Zhang, X. LinDA: linear models for differential abundance analysis of microbiome compositional data. *Genome Biol* **23**, (2022).

12. Anderson, M. J., Ellingsen, K. E. & McArdle, B. H. Multivariate dispersion as a measure of beta diversity. *Ecol Lett* **9**, 683–693 (2006).

13. Office for National Statistics. Population estimates for the UK, England and Wales, Scotland and Northern Ireland. *Statistical bulletin* https://www.ons.gov.uk/peoplepopulationandcommunity/populationandmigration/populationestimates/bulletins/annualmidyearpopulationestimates/mid2017 (2017).

14. Kingston, A. *et al.* Is late-life dependency increasing or not? A comparison of the Cognitive Function and Ageing Studies (CFAS). *The Lancet* **390**, 1676–84 (2017).

15. Welch, A. A. Nutritional influences on age-related skeletal muscle loss. *Proceedings of the Nutrition Society* **73**, 16–33 (2014).

16. Rejc, E. *et al.* Loss of maximal explosive power of lower limbs after 2 weeks of disuse and incomplete recovery after retraining in older adults. *The Journal of Physiology* **596**, 647–665 (2018).

17. Houston, D. K. *et al.* Dietary protein intake is associated with lean mass change in older, community-dwelling adults: the Health , Aging, and Body Composition (Health ABC) Study. *Am J Clin Nutr 2008;87:150–5.* **87**, 150–155 (2008).

18. Ni Lochlainn, M., Bowyer, R. & Steves, C. Dietary Protein and Muscle in Aging People: The Potential Role of the Gut Microbiome. *Nutrients* **10**, 929 (2018).

19. Deutz, N. E. P. *et al.* Protein intake and exercise for optimal muscle function with aging: Recommendations from the ESPEN Expert Group. *Clin Nutr. 2014* **33**, 929–936 (2014).

20. Tieland, M., Borgonjen-Van Den Berg, K. J., Van Loon, L. J. C. & De Groot, L. C. P. G. M. Dietary protein intake in community-dwelling, frail, and institutionalized elderly people: Scope for improvement. *Eur J Nutr* **51**, 173–179 (2012).

21. Vigotsky, A. D., Schoenfeld, B. J., Than, C. & Brown, J. M. Methods matter: The relationship between strength and hypertrophy depends on methods of measurement and analysis. *PeerJ* (2018) doi:10.7717/peerj.5071.

22. Biagi, E. *et al.* Through Ageing, and Beyond: Gut Microbiota and Inflammatory Status in Seniors and Centenarians. *PLoS ONE* **5**, e10667 (2010).

23. Ridaura, V. K. *et al.* Gut microbiota from twins discordant for obesity modulate metabolism in mice. *Science* **341**, 1241214 (2013).

24. Smith, P. *et al.* Regulation of Life Span by the Gut Microbiota in The Short-Lived African Turquoise Killifish. *bioRxiv* 120980 (2017) doi:10.1101/120980.

25. Buigues, C. *et al.* Effect of a Prebiotic Formulation on Frailty Syndrome: A Randomized, Double-Blind Clinical Trial. *International journal of molecular sciences* **17**, (2016).

26. Theou, O. *et al.* Can a Prebiotic Formulation Reduce Frailty Levels in Older People? *The Journal of frailty & aging* **8**, 48–52 (2019).

27. Walker, S. N. *et al.* Maintenance of activity and eating change after a clinical trial of tailored newsletters with older rural women. *Nurs Res* **59**, 311–321 (2010).

28. Cesari, M. *et al.* Added Value of Physical Performance Measures in Predicting Adverse Health-Related Events: Results from the Health, Aging, and Body Composition Study. *J Am Geriatr Soc* **57**, 251–259 (2009).

29. Harvey, N. C. *et al.* Measures of Physical Performance and Muscle Strength as Predictors of Fracture Risk Independent of FRAX, Falls, and aBMD: A Meta-Analysis of the Osteoporotic Fractures in Men (MrOS) Study. *Journal of Bone and Mineral Research* **33**, 2150–2157 (2018).

30. RStudio Team. RStudio: Integrated Development for R. *RStudio, Inc., Boston, MA* (2015).

31. Welch, A. A. Nutritional influences on age-related skeletal muscle loss. *Proceedings of the Nutrition Society* **73**, 16–33 (2014).

32. Deutz, N. E. P. *et al.* Protein intake and exercise for optimal muscle function with aging: Recommendations from the ESPEN Expert Group. *Clin Nutr. 2014* **33**, 929–936 (2014).

33. Biagi, E. *et al.* Through Ageing, and Beyond: Gut Microbiota and Inflammatory Status in Seniors and Centenarians. *PLoS One* **5**, e10667 (2010).

34. Ridaura, V. K. *et al.* Gut microbiota from twins discordant for obesity modulate metabolism in mice. *Science (1979)* **341**, 1241214 (2013).

35. E9 Expert Working Group. ICH Harmonised Tripartite Guideline. Statistical principles for clinical trials. International Conference on Harmonisation. *Stat Med* **18**, 1905–42 (1999).

36. Ni Lochlainn, M. *et al.* The PROMOTe study: targeting the gut microbiome with prebiotics to overcome age-related anabolic resistance: protocol for a double-blinded, randomised, placebo-controlled trial. *BMC Geriatr* **21**, 407 (2021).

37. Bowyer, R. C. E. *et al.* Use of dietary indices to control for diet in human gut microbiota studies. *Microbiome* **6**, 77 (2018).

38. Searle, S. D., Mitnitski, A., Gahbauer, E. A., Gill, T. M. & Rockwood, K. A standard procedure for creating a frailty index. *BMC Geriatr* **8**, 24 (2008).

39. Cox, N. J. *et al.* The composition of the gut microbiome differs among community dwelling older people with good and poor appetite. *J Cachexia Sarcopenia Muscle* **12**, 368–377 (2021).

40. Wilson, M.-M. G. M. G. *et al.* Appetite assessment: simple appetite questionnaire predicts weight loss in community-dwelling adults and nursing home residents. *Am J Clin Nutr* **82**, 1074–1081 (2005).

41. Ipaq.ki.se. *Guidelines for Data Processing and Analysis of the International Physical Activity Questionnaire (IPAQ)-Short Form*. www.ipaq.ki.se. (2004).

42. CRAIG, C. L. *et al.* International Physical Activity Questionnaire: 12-Country Reliability and Validity. *Med Sci Sports Exerc* **35**, 1381–1395 (2003).

43. Cruz-Jentoft, A. J. *et al.* Sarcopenia: revised European consensus on definition and diagnosis. *Age Ageing* **48**, 16–31 (2019).

44. Black, A. E. The sensitivity and specificity of the Goldberg cut-off for EI:BMR for identifying diet reports of poor validity. *Eur J Clin Nutr* **54**, 395–404 (2000).

45. Black, A. E. Critical evaluation of energy intake using the Goldberg cut-off for energy intake:basal metabolic rate. A practical guide to its calculation, use and limitations. *Int J Obes* **24**, 1119–1130 (2000).

46. Roza, A. M. & Shizgal, H. M. The Harris Benedict equation reevaluated: resting energy requirements and the body cell mass. *Am J Clin Nutr* **40**, 168–182 (1984).

47. Dietary reference values for food energy and nutrients for the United Kingdom. Report of the Panel on Dietary Reference Values of the Committee on Medical Aspects of Food Policy - PubMed. https://pubmed.ncbi.nlm.nih.gov/1961974/.

48. RStudio Team. RStudio: Integrated Development for R. *RStudio, Inc., Boston, MA* (2015).

49. Kahan, B. C., Jairath, V., Doré, C. J. & Morris, T. P. The risks and rewards of covariate adjustment in randomized trials: an assessment of 12 outcomes from 8 studies. *Trials* **15**, 139 (2014).

50. White, I. R., Horton, N. J., Carpenter, J., Statistics, r. i. m. a. s. & Pocock, S. J. Strategy for intention to treat analysis in randomised trials with missing outcome data. *BMJ* **342**, d40–d40 (2011).

<u>Appendix 1 – Information with regards to Safety Reporting in Non-CTIMP Research</u>

	Who	When	How	To Whom
**SAE**	Chief Investigator	-Report to Sponsor within 24 hours of learning of the event<br><br>-Report to the MREC within 15 days of learning of the event	SAE Report form for Non-CTIMPs, available from NRES website.	Sponsor and MREC
**Urgent Safety Measures**	Chief Investigator	Contact the Sponsor and MREC Immediately<br><br>Within 3 days	By phone<br><br>Substantial amendment form giving notice in writing setting out the reasons for the urgent safety measures and the plan for future action.	Main REC and Sponsor<br><br>Main REC with a copy also sent to the sponsor. The MREC will acknowledge this within 30 days of receipt.
**<u>Progress Reports</u>**	Chief Investigator	Annually (starting 12 months after the date of favourable opinion)	Annual Progress Report Form (non-CTIMPs) available from the NRES website	Main REC
**<u>Declaration of the conclusion or early termination of the study</u>**	Chief Investigator	Within 90 days (conclusion)<br><br>Within 15 days (early termination)<br><br>*The end of study should be defined in the protocol*	End of Study Declaration form available from the NRES website	Main REC with a copy to be sent to the sponsor
**<u>Summary of final Report</u>**	Chief Investigator	Within one year of conclusion of the Research	No Standard Format<br><br>However, the following Information should be included:	Main REC with a copy to be sent to the sponsor

			Where the study has met its objectives, the main findings and arrangements for publication or dissemination including feedback to participants	

21 Statistical Analysis Plan

Statistical Analysis Plan

Dietary Protein and Skeletal Muscle in Older Twins - Targeting the Gut Microbiome to Overcome Anabolic Resistance:

The PROMOTe Study

ClinicalTrials.gov identifier:	NCT04309292
Author:	Dr Mary Ni Lochlainn
Version:	1.1
Date of PDF-ing	09/03/2022

Table of Contents

1 Abbreviations 73
2 Introduction 74
  2.1 Background and Rationale 74
  2.2 Study Objectives 74
3 Study Methods 75
  3.1 Trial Design 75
    3.1.1 Figure 1: Trial Design 76
  3.2 Randomisation/Allocation Concealment/Blinding Procedures 76
  3.3 Sample size calculation 76
4 Trial Population 77
  4.1 Screening data and eligibility criteria 77
  4.2 Presentation of baseline characteristics 77
    4.2.1 Table 1: Baseline Characteristics 77
  4.3 Figure 2: Participant flow diagram 79
5 Analysis Populations 80
  5.1 Adherence 80
  5.2 Figure 3: Analysis Populations 81
  5.3 Subgroups 81
6 Hypothesis Testing 82
  6.1 Primary Outcome 82
  6.2 Secondary Outcomes 82
  6.3 Safety Outcomes 82
7 Statistical Analysis 83
  7.1 Descriptive Statistics 83
  7.2 Main Analyses 83
    7.2.1 Primary Outcome 84
    7.2.2 Secondary Outcomes 84
  7.3 Sensitivity Analyses 85
  7.4 Validation Analyses .... **Error! Bookmark not defined.**
  7.5 Missing data 85
    7.5.1 Missing outcome data 85
    7.5.2 Missing baseline covariate data 86
  7.6 Planned tables and figures 86
    7.6.1 Table 2: Planned tables and figures 87
8 References 88

1. Abbreviations

BMI	Body Mass Index
BMR	Basal Metabolic Rate
CI	Confidence interval
DZ	Dizygotic
EWGSOP2	European Working Group for Sarcopenia of Older Persons 2
FI	Frailty Index
Fid	Family ID
HEI	Healthy Eating Index
ICH	International Conference on Harmonisation of Technical Requirements for Registration of Pharmaceuticals for Human Use
IPAQ	International Physical Activity Questionnaire
IQR	Inter-quartile range
ITT	Intention to Treat
KCTU	King's College London Clinical Trials Unit
MAR	Missing at random
MCAR	Missing completely at random
MNAR	Missing not at random
MPB	Muscle protein breakdown
MPS	Muscle protein synthesis
MZ	Monozygotic
PP	Per Protocol
SAE	Serious adverse event
SAP	Statistical Analysis Plan
SD	Standard deviation
SNAQ	Simplified Nutritional Appetite Questionnaire
SPPB	Short Physical Performance Battery

2 Introduction

Background and Rationale

The age of populations worldwide is increasing. This comes with an increase in age-related conditions and time spent living with age-related morbidity [14]. We lose skeletal muscle as we age. Protein in diet is crucial for maintaining skeletal muscle health, however several factors can lead to reduced protein intake in older age, including social isolation, dysphagia, slower gastric emptying etc.

In addition to taking less protein in their diet, research has shown that older adults display anabolic resistance to protein intake, a blunted responsiveness of older muscle in terms of muscle protein synthesis, compared with younger adults [31]. This has led to a higher daily intake of 1-1.3g/kg/day being recommended by experts [32]. The UK Reference Nutrient Intake for adults is 0.8 g/kg/day.

Skeletal muscle mass is regulated by the processes of muscle protein synthesis and breakdown (MPS and MPB). MPS rates are largely controlled by responsiveness to anabolic stimuli, e.g., consumption of food, and physical activity. Catabolic stressors include illness, physical inactivity, and inflammation, of which older people tend to have higher rates. The aetiology of anabolic resistance is complex, involving aging physiology and physical inactivity.

The gut microbiome is composed of bacteria, archaea, viruses, and eukaryotic microbes that reside in the gut. Its role in maintaining healthy physiology is a rapidly evolving field of enquiry. With age, the resilience of the gut microbiome is reduced, as it becomes more vulnerable to lifestyle changes, with changed species richness and increased inter-individual variability [33].

The potential of the gut microbiota to alter physiology has been shown by landmark animal faecal transplant studies, which have demonstrated body composition changes in the recipient reflective of the donor's phenotype [34]. This highlights the role of microbiota in characterising metabolic phenotypes. Several different mechanisms have been proposed for anabolic resistance, and the gut microbiome plays a role in many of these. Examples include protein digestion and absorption, gut barrier function, and inflammation [18]. This study aims to assess whether the gut microbiome modulates anabolic resistance, therefore presenting a malleable therapeutic target for prevention and reversal of muscle loss with age.

The International Conference on Harmonisation of Technical Requirements for Registration of Pharmaceuticals for Human Use (ICH) recommends that data from clinical trials be analysed according to a pre-specified statistical analysis plan (SAP) [35]. This SAP has been developed and finalised without knowledge of study arm allocation or study results and all analysis will be carried out blinded.

Study Objectives

The current study's objective is to investigate whether prebiotic food supplementation in combination with protein supplementation improves muscle strength in older ambulant community dwelling individuals.

Specific Aim 1: carry out an interventional dietary study to test whether modulation of the gut microbiome (via prebiotic food supplements), in addition to protein supplementation, can improve skeletal muscle function (muscle strength – measured using chair-rise time) versus protein supplementation alone.

Specific Aim 2: To deliver a wealth of data, including effect sizes, on specific causal links between protein supplementation, prebiotic food supplementation, changes in the gut microbiome composition, changes in the metabolomic profile and changes in skeletal muscle function of an individual.

Additional objective: evaluate remotely delivered trial – validate measures taken remotely

In light of the COVID-19 global pandemic and the resulting restrictions on travel, this protocol has been adapted such that all 'visits' will be carried out online, via video teleconferencing software. Participants will take part from home, with the study visits taking place over video teleconferencing. The landscape of research will change drastically in the post COVID-19 era and research carried out remotely, utilising technology and innovative techniques, will become both more common and necessary. A subset of the study population will be invited to the department for repeat physical measures for validation purposes, which will be optional to them.

3. Study Methods

Trial Design

The trial design is described in detail in previously published study protocol (Ni Lochlainn et al., 2021) and briefly described here.

The study is a randomised, placebo controlled, double blinded, intervention study in which participants were grouped into two different arms. One arm received protein food supplement plus prebiotic food supplement and the other arm received protein food supplement plus placebo (maltodextrin). Supplements were posted to participants in pre-mixed identical sachets, which they were advised to take daily for 12 weeks, in a glass of water or other drink. Participants in both groups were also advised to undertake resistance exercises. All participants had a study visit carried out via video teleconferencing software at the beginning and end of the study. They also completed questionnaires, food diaries and cognitive testing remotely at the beginning and end of the study. Lastly the participants also collected biological samples (urine, stool, saliva, and capillary blood) and posted these to the department at the beginning and end of the study. A subset of participants also completed an additional study visit in-person at the department for validation measures.

Randomisation was used to control for equal distribution of key characteristics that may confound between group comparisons. Participants were randomised as twin pairs, with one twin from the pair in the prebiotic food supplement arm and one twin from the pair in the placebo arm. Twin pairs are matched for age and often for sex, early environment, baseline genetic sequence, socioeconomic status etc., proving a strong model for detecting changes while adjusting for both observed and unobserved confounding.

i. Figure 1: Trial Design

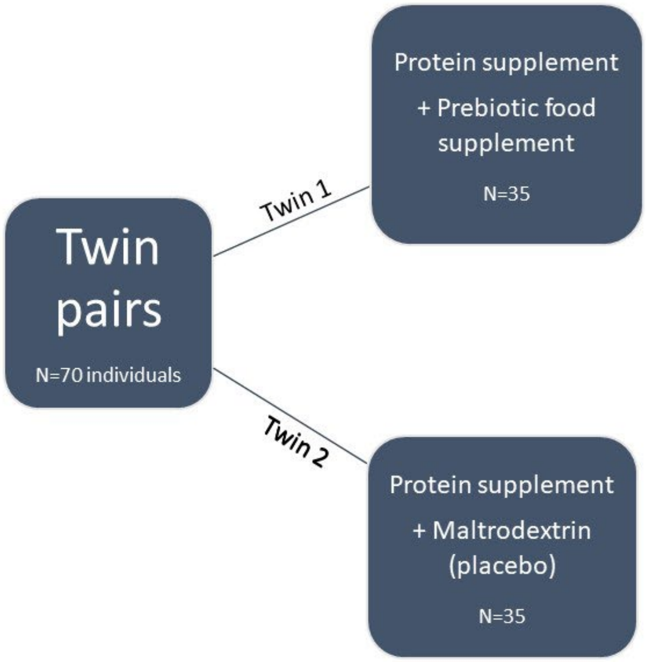

Randomisation/Allocation Concealment/Blinding Procedures

Participants will undergo computer generated block randomisation in twin pairs by the King's College London Clinical Trials Unit (KCTU). They will be randomised as twin pairs, with one twin in each arm. Researchers and participants will be blinded as to which arm each participant is in.

On completion of the data collection, the KCTU will issue a blinded list denoting what arm each participant is in (Arm 1; Arm 2), but without revealing which arm is the intervention and which the placebo, in order to facilitate blinded analysis to be carried out. Full unblinding will not take place until data analysis is complete. Until that point, only the KCTU will have access to the unblinded information.

Sample size calculation

From our existing data, we have observed that chair-rise time is to a good approximation log normal, with log10(time) having a SD of 0.126. We consider a relative reduction in average time of 20% to be both clinically important and plausible. Based on these figures, we would need complete data on 30 subjects per group (60 in total) for 80% power. Allowing for 20% dropouts, we would need 70 (35 per group) recruited. Based on the numbers we plan to recruit for the study, we estimate a recruitment time period of 18 months.

We have estimated a powered sample size based on other studies using chair-rise time [27–29], however we note that no study has looked at this in the context of protein and/or prebiotic food supplementation and therefore may not be accurate.

4. Trial Population

a. Screening data and eligibility criteria

Participants will be recruited from the TwinsUK databases and invited to attend a virtual visit using video teleconferencing. A subset will later be invited to the department in St Thomas' Hospital to repeat the physical performance measures. Those who are aged 60 years or older and who have a dietary protein intake of <1.3g/kg/day on their most recent food frequency questionnaire will be contacted and invited for screening.

b. Presentation of baseline characteristics

The most recent available data within the TwinsUK cohort will be used for the frailty and healthy eating indices, as described previously [37,38]. Questionnaire data will be used to calculate the SNAQ score for each participant at both time points [39,40]. Likewise, questionnaire data will be used to generate IPAQ score and MET minutes using the standard approach [41,42].

i. Table 1: Baseline Characteristics

Characteristic	Measure	Detail	Display
Age	Years	continuous	mean,(SD)
Sex	Male, female	binary	n,(%)
Zygosity	Monozygotic, dizygotic	binary	n,(%)
Smoking status	Never, Former, Current	categorical	n,(%)
Household Income	Declined to answer, low, middle, high	categorical	n,(%)
Education	Low, middle, high	categorical	n,(%)
Alcohol Intake	Never, less than weekly, at least weekly	categorical	n,(%)
Height	Metres (m)	continuous	mean,(SD)
Weight	Kilograms (Kg)	continuous	mean,(SD)
BMI	Kg/m^2	continuous	mean,(SD)
Protein Intake	g/day	continuous	mean,(SD)
Protein Intake/weight	g/kg/day		
Energy Intake	Kcal/day	continuous	mean,(SD)
Physical Activity - IPAQ	Low, Moderate, High	categorical	n,(%)
IPAQ MET minutes	Minutes	continuous	mean,(SD)
Healthy Eating Index	Score	continuous	mean,(SD)
Frailty Index	Score	continuous	mean,(SD)

Appetite – SNAQ	Score	continuous	mean,(SD)
Gait speed	Metres/second	continuous	mean,(SD)
Chair rise time	Seconds	continuous	mean,(SD)
Grip strength	Kilograms	continuous	mean,(SD)
Balance score	Score	continuous	mean,(SD)
SPPB score	Score	continuous	mean,(SD)
Cognition	Scores for 5 main tests	continuous	mean,(SD)

c. *Figure 2: Participant flow diagram*

i.

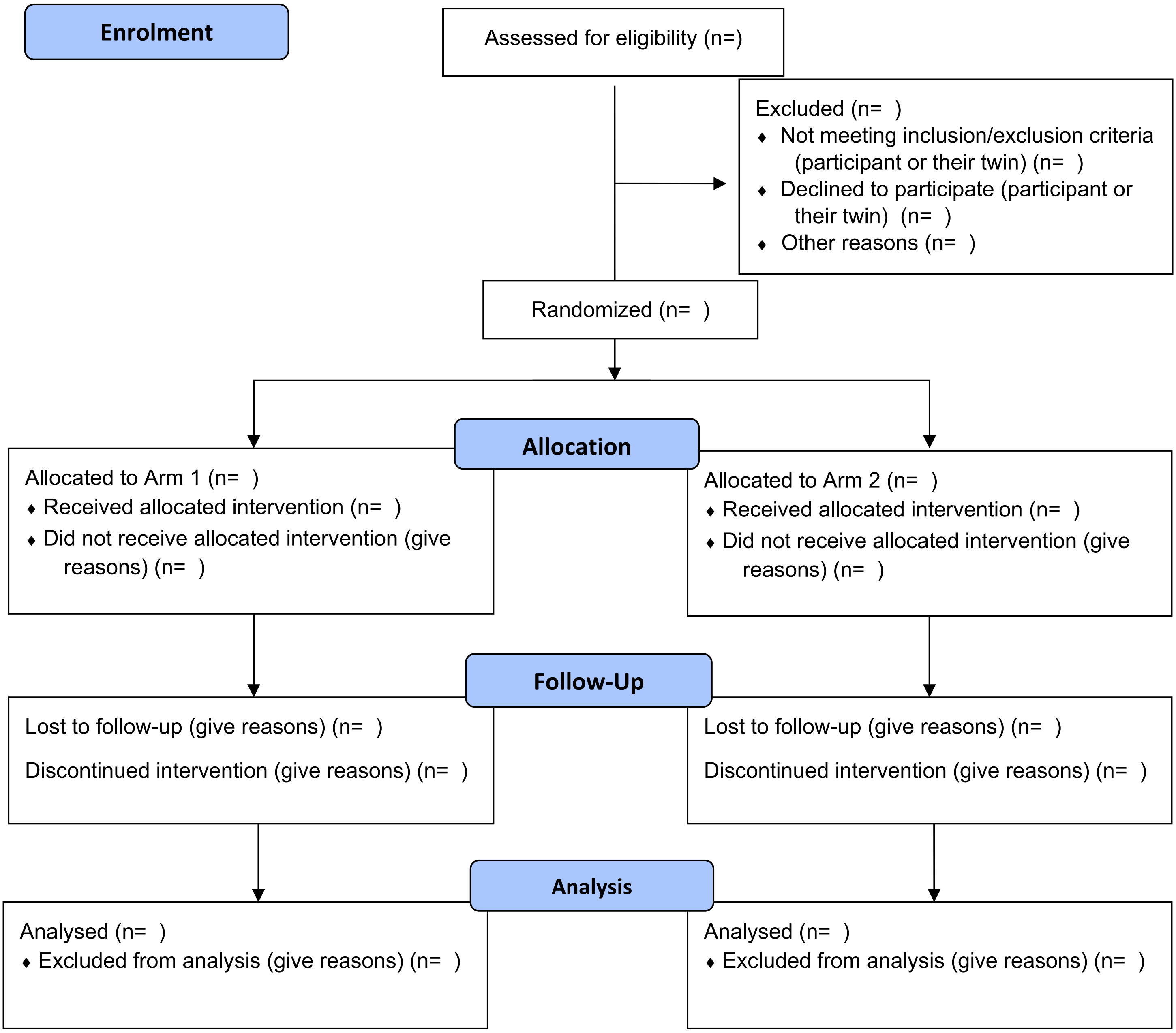

5. Analysis Populations

Intention-to-treat (ITT) will include all randomised study subjects. This will be seen as the primary population for the analysis.

Per Protocol (PP) will include all randomised study subjects completing the whole study period (complete cases). For a specific analysis, study subjects with missing data on any of the variables in the model will be excluded from the analysis. Analyses of this population is seen as a sensitivity analysis to investigate whether conclusions are sensitive to assumptions regarding the pattern of missing data.

In our study, those who have not completed both study visits will be excluded from PP analysis. In addition, those who have lower than 50% adherence to the study intervention will also be excluded.

a. Adherence

Adherence will be assessed by counting the number of sachets of supplement remaining at the final visit and dividing this into the total number of sachets issued to the participant. This will create a new data point X for all participants. An adherence variable will be created with the formula 100-X. In addition, a categorical variable will be created of low (<50%), medium (51-75%) and high (>75%) adherence.

While the planned intervention period is 12 weeks, this will vary by a number of days for each participant. In order to account for this variation, the number of days between the first and second visit will be calculated. Appropriate adjustments will be made for any participants who have additional days in their intervention period.

b. Figure 3: Analysis Populations

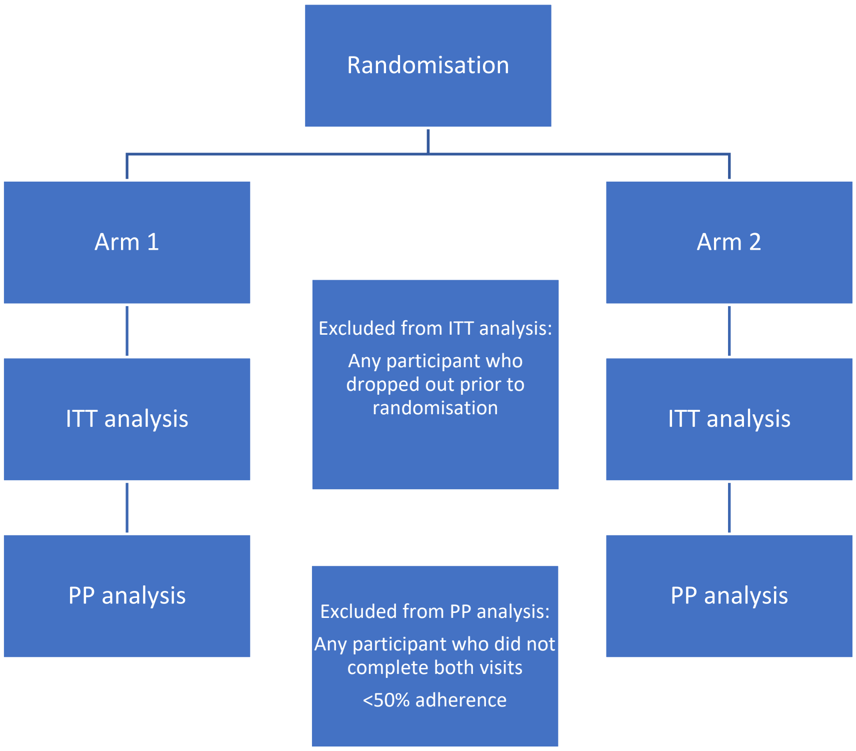

c. Subgroups

Several subgroups will be formed based on the following characteristics:

1. Sex
2. Age
3. Zygosity
4. Protein intake at baseline
5. Exercise level (IPAQ MET minutes) at baseline
6. Frailty index at baseline
7. SPPB at baseline
8. Gut microbiome alpha diversity at baseline
9. Gut microbiome beta diversity at baseline
10. Gut microbiome responders, defined as those who had changes in the function and/or composition of their gut microbiota from baseline to study end

The primary outcome and relevant secondary outcomes will be re-assessed in these subgroups. It is recognised that the study is not powered for these analyses. Linear mixed models as described in 7.2.1 and 7.2.2 will be carried out in each subgroup. Finally, some subgroups will also be used for group-wide analyses [See Section 5.4], namely protein intake at baseline, and frailty index at baseline.

d. Overall Improvement

In addition to analysing the primary and secondary outcomes by arm, group-wide comparisons will be carried out for the following variables of interest: weight, BMI, SPPB

score, balance score, grip strength, chair rise time, gait speed, IPAQ MET minutes/week, IPAQ score, SNAQ score, CANTAB cognitive test results and serum metabolites.

Paired tests will be carried out to assess for differences between baseline and study end point, two sample t-tests for continuous variables (normally distributed), Mann-Whitney test for non-normally distributed continuous variables, and Pearson's chi-squared tests for categorical variables.

While there is no control group for these analyses, these will tell us whether the protein and exercise intervention that all twins will receive was beneficial for the aforementioned variables of interest, irrespective of gut microbiome modulation with a prebiotic food supplement. This analysis will also be repeated using two subgroups of interest, protein intake at baseline, and frailty index at baseline.

6. Hypothesis Testing

a. Primary Outcome

The primary aim of the study is to test whether modulation of the gut microbiome using a prebiotic food supplement, in addition to protein supplementation, can improve muscle strength versus protein supplementation alone. Thus, the primary efficacy outcome is a variable which measures muscle strength: chair rise time, the time it takes to rise from a standard chair five times, without using one's arms. This was chosen based on the EWGSOP2 guidance for measuring muscle strength in the context of sarcopenia research [43].

Primary outcome: change in chair rise time, operationalised by using chair rise time measured at end of study, and including baseline chair rise time in the statistical model

Null Hypothesis: there is no difference in the change in chair rise time between study arm 1 (prebiotic + protein) and study arm 2 (placebo + protein)

b. Secondary Outcomes

Secondary outcomes include:

- Grip strength
- Short physical performance battery (SPPB) score
- IPAQ - physical activity questionnaire – either overall score or MET minutes/week
- Appetite - SNAQ questionnaire
- Cognition (as measured by CANTAB battery)
- Gut microbiome measures
- Serum metabolites

Null Hypotheses: there is no differences in the change of each of the above measures between study arm 1 and study arm 2. [See Section 7.2.2 for further detail on analysis plan].

i. Cognition: Factor Analysis

Key variables will be chosen from the results of each of the tests of the CANTAB cognitive battery. A factor analysis will be carried out using the chosen variables. The same factor

loadings used for the baseline cognitive test results, will be applied to the end of study test results, leaving a baseline factor score and a study-end factor score. These factor scores will be used in the analyses laid out in Section 7.2.1.

c. Safety Outcomes

All safety outcomes will be reviewed, collated, and reported where relevant. These include adverse events, serious adverse events (SAEs), hospitalisations or other significant medical events.

Adverse effects will be defined as present or absent (coded 1 and 0 respectively) for each participant. Participants who dropped out prior to receiving any of the intervention will be excluded. Each binary safety outcome will be analysed by treatment group using a chi-square test or Fisher exact test. The likelihood of an SAE being associated with the study intervention will be rated as none, unlikely, possible, or probable. Frequencies and percentages of any adverse events in each study arm will be calculated.

7. Statistical Analysis

a. Descriptive Statistics

All outcomes will be presented using descriptive statistics; normally distributed data by the mean and standard deviation (SD) and skewed distributions by the median and interquartile range (IQR). Binary and categorical variables will be presented using counts and percentages. P values of ≤0.05 will be considered sufficient to reject the null hypothesis.

Outliers will be assessed by examining plots and checked for possible erroneous entry. Where necessary, results with and without outliers will be compared to assess for discrepancies in the results, and this will be discussed if found to be discrepant.

i. Food diary data

The food diary data will be presented in tables, by sex. Paired tests will be carried out to assess for differences between baseline and study end point, two sample t-tests for continuous variables (normally distributed), and Mann-Whitney test for non-normally distributed continuous variables.

Over and under reporting will be assessed using the Goldberg energy intake to BMR (basal metabolic rate) ratio method [44,45]. BMR will be calculated using the revised Harris-Benedict equation [46], and multiplied by an adjustment factor for physical activity [47]. A ratio of this adjusted BMR to energy intake (kcals/day) will be generated. Over and under reporters will be identified as those who are two standard deviations above or below this ratio. If over an/or under reporters are identified, an additional dietary table will be presented, with these participants excluded.

b. Main Analyses

Paired tests will be carried out to assess for differences between each arm at baseline; two sample t-tests for continuous variables (normally distributed), Mann-Whitney test for non-normally distributed continuous variables, and Pearson’s chi-squared tests for categorical variables.

All primary statistical analyses will be unadjusted. Subsequent analyses carried out will be adjusted for relevant covariates which are known or suspected *a priori* to be associated with the outcome, as described henceforth. Stata and RStudio [48] will be used for all statistical analysis.

i. Primary Outcome

The primary analysis will compare intervention groups (arm 1 vs arm 2; blinded) on their change in chair rise time at 12 weeks using a linear mixed model. Chair rise time at 12 weeks will be the dependent variable. Twin clustering will be considered as random effects, treatment group as fixed effect. Baseline value of chair rise time will be included as a covariate [See: [49]]. If, despite randomisation, there is a difference between the two arms for a specific baseline characteristic, for example age, then age will be included as a covariate in the linear mixed models. The estimated difference in mean change from baseline to 12 weeks and the corresponding 95 % confidence interval (CI) will be presented.

The nature of the design of this study, with randomisation by twin pair and therefore clustering within the data will be taken into consideration by using both twin pair identifier (fid) and zygosity as random effects.

The number of participants who experience a 20% reduction in their chair rise time, as has been deemed clinically significant in previous studies (See Section 3.3), will be presented. We acknowledge that none of these studies used an intervention targeting the gut microbiota, however none such studies were available, making these the closest benchmark available.

ii. Secondary Outcomes

Secondary analyses will include linear mixed effects regression models to investigate associations between the change in each of the below variables, adjusting for covariates where relevant.

- Grip strength
- Short physical performance battery (SPPB) score
- IPAQ - physical activity questionnaire – either overall score or MET minutes/week
- Appetite - SNAQ questionnaire
- Cognition (as measured by CANTAB battery)
- Gut microbiome measures – function and composition (from faecal samples) [See Section 7.2.2.1 for further detail].
- Serum metabolites

The change in grip strength, SPPB score, IPAQ score, FI, cognition, and SNAQ will be analysed using the same method as for the primary outcome, including usage of the baseline value for the actual factor as a covariate.

An exploratory analysis will be carried out to assess for any differences in serum metabolite profile between the two arms of the study. Linear mixed models will be used as described in Section 7.2.1 using the metabolite at study end as the dependent variable and including the baseline level as a covariate. Analyses will be adjusted for multiple testing to control the overall type 1 error rate.

1. Gut Microbiome Function and Composition

Stool samples will be collected from all participants at the beginning and end of the study, via post. The DNA extraction will be carried out in our own laboratory, and then the extracted DNA samples will be sent to an external microbiome sequency and bioinformatic company, who will carry out high-resolution shotgun metagenomics microbiome analysis of all samples. This includes full profiling of microbiota present (composition), functional species group association analysis, as well as measures of alpha and beta diversity for each individual sample.

Once the above information is available, statistical analysis will assess microbiota differences between the two study arms and between twin pairs. Microbiome composition will be analysed with respect to (1) species diversity, (2) compositional differences, (3) functional differences and (4) differences in abundance of taxa. All analysis will take a hierarchical approach; crude models assessing just the variable of interest (prebiotic supplementation), and models adjusted for key biological (age, sex, exercise etc..) and technical covariates (e.g., differences in library size) will be applied.

Differences in species diversity and richness will be compared between each study arm in regression analysis in crude and adjusted models. Distance matrixes characterise the inter-individual differences in microbiota composition; ordination analyses and permutational multivariate analysis of variance (PERMANOVA) will be used to understand differences in composition between each study arm.

ASVs will be collapsed to family, order, and phylum levels, with hierarchical models, adjusted for each potential mediator individually, then fully adjusted. All models will be adjusted for multiple testing.

All twins within each study arm will be used in paired and unpaired tests to assess if microbiota dissimilarity increased with differences in muscle strength, taking relevant covariates (including diet [HEI], BMI and frailty index) into consideration.

c. Sensitivity Analyses

If necessary, sensitivity analyses will be conducted to assess the impact of poor adherence (self-reported), missing data, outliers and/or unexpected imbalances between the two groups at baseline. These analyses will be compared to the primary analyses to assess any differences in conclusions.

d. Missing data

i. Missing outcome data

We will follow a four-point framework for dealing with incomplete observations which will allow the correct method to be chosen and subsequently implemented [50].

1. Attempt to follow up all randomised participants, even if they withdraw from allocated treatment
2. Perform a main analysis of all observed data that is valid under a plausible assumption about the missing data. Specifically, we will assume data is missing at random (MAR). Under this assumption, imbalances between treatment groups due to dropout can be corrected by appropriate multiple regression models.
3. Perform a sensitivity analysis to explore the effect of departures from the assumption made in the main analysis. The MNAR (missing not at random) analysis will use the method of White et al. (2011) [50].

4. Account for all randomised participants, at least in the sensitivity analyses

This framework highlights the importance of using plausible assumptions with regards to the nature of the missing data. These assumptions will then be tested using appropriate sensitivity analyses on observed data using complete cases analysis. For the purpose of the main analysis, we will assume that missing data is missing at random, and the effect of the intervention is the same in those with and without the observations. Furthermore, we will check whether there is an imbalance or is similar the percentage of missing data within each treatment allocation.

ii. Missing baseline covariate data

Participants with outcome data will not be excluded if they are missing baseline covariate data, in order to satisfy the principles of an ITT approach. Thus, for missing data in this category, mean imputation techniques will be used. Alternatively for some variables, the most recent value from the longitudinal TwinsUK cohort data will be imputed, if these is deemed to be within a sensible period of time. The timeframe will vary according to the variable in question. For example, education level is unlikely to change over a 5-year period in this age group. While mean imputation can introduce bias in statistical estimates, the randomisation at baseline ensures baseline variables are independent of which arm the participant is in. All imputation of unknowns with approximated values will be decided on before unblinding.

e. Planned tables and figures

i. Table 2: Planned tables and figures

**Table/Figure**	**Title**
Figure 1	Participant Flow Diagram (see 4.3)
Table 1	Comparison of participants to those who declined/were ineligible
Table 2	Baseline Characteristics, by study arm (see 4.2.1)
Table 3	Group wide comparisons between baseline and study end point
Table 4	Dietary Intakes at baseline and study end
Table 5	Cognition Factor Analysis
Table 6	Adverse events, dropouts, compliance
Table 7	Linear Mixed models for primary and secondary outcomes
Table 8	Sensitivity Analyses
Table 9	Subgroup Analyses
Table 10	Validation Analyses

8. References

1. Pasolli, E. *et al.* Extensive Unexplored Human Microbiome Diversity Revealed by Over 150,000 Genomes from Metagenomes Spanning Age, Geography, and Lifestyle. *Cell* **176**, 649-662.e20 (2019).

2. Nielsen, H. B. *et al.* Identification and assembly of genomes and genetic elements in complex metagenomic samples without using reference genomes. *Nat Biotechnol* **32**, 822–828 (2014).

3. Parks, D. H., Imelfort, M., Skennerton, C. T., Hugenholtz, P. & Tyson, G. W. CheckM: assessing the quality of microbial genomes recovered from isolates, single cells, and metagenomes. *Genome Res* **25**, 1043–1055 (2015).

4. Langmead, B. & Salzberg, S. L. Fast gapped-read alignment with Bowtie 2. *Nat Methods* **9**, 357–359 (2012).

5. Schubert, M., Lindgreen, S. & Orlando, L. AdapterRemoval v2: rapid adapter trimming, identification, and read merging. *BMC Res Notes* **9**, 88 (2016).

6. Li, H. & Durbin, R. Fast and accurate short read alignment with Burrows–Wheeler transform. *Bioinformatics* **25**, 1754–1760 (2009).

7. Huerta-Cepas, J. *et al.* Fast Genome-Wide Functional Annotation through Orthology Assignment by eggNOG-Mapper. *Mol Biol Evol* **34**, 2115–2122 (2017).

8. Kanehisa, M. KEGG: Kyoto Encyclopedia of Genes and Genomes. *Nucleic Acids Res* **28**, 27–30 (2000).

9. Vieira-Silva, S. *et al.* Species–function relationships shape ecological properties of the human gut microbiome. *Nat Microbiol* **1**, 16088 (2016).

10. Valles-Colomer, M. *et al.* The neuroactive potential of the human gut microbiota in quality of life and depression. *Nat Microbiol* **4**, 623–632 (2019).

11. Zhou, H., He, K., Chen, J. & Zhang, X. LinDA: linear models for differential abundance analysis of microbiome compositional data. *Genome Biol* **23**, (2022).

12. Anderson, M. J., Ellingsen, K. E. & McArdle, B. H. Multivariate dispersion as a measure of beta diversity. *Ecol Lett* **9**, 683–693 (2006).

13. Office for National Statistics. Population estimates for the UK, England and Wales, Scotland and Northern Ireland. *Statistical bulletin* https://www.ons.gov.uk/peoplepopulationandcommunity/populationandmigration/populationestimates/bulletins/annualmidyearpopulationestimates/mid2017 (2017).

14. Kingston, A. *et al.* Is late-life dependency increasing or not? A comparison of the Cognitive Function and Ageing Studies (CFAS). *The Lancet* **390**, 1676–84 (2017).

15. Welch, A. A. Nutritional influences on age-related skeletal muscle loss. *Proceedings of the Nutrition Society* **73**, 16–33 (2014).

16. Rejc, E. *et al.* Loss of maximal explosive power of lower limbs after 2 weeks of disuse and incomplete recovery after retraining in older adults. *The Journal of Physiology* **596**, 647–665 (2018).

17. Houston, D. K. *et al.* Dietary protein intake is associated with lean mass change in older, community-dwelling adults: the Health , Aging, and Body Composition (Health ABC) Study. *Am J Clin Nutr 2008;87:150–5.* **87**, 150–155 (2008).

18. Ni Lochlainn, M., Bowyer, R. & Steves, C. Dietary Protein and Muscle in Aging People: The Potential Role of the Gut Microbiome. *Nutrients* **10**, 929 (2018).

19. Deutz, N. E. P. *et al.* Protein intake and exercise for optimal muscle function with aging: Recommendations from the ESPEN Expert Group. *Clin Nutr. 2014* **33**, 929–936 (2014).

20. Tieland, M., Borgonjen-Van Den Berg, K. J., Van Loon, L. J. C. & De Groot, L. C. P. G. M. Dietary protein intake in community-dwelling, frail, and institutionalized elderly people: Scope for improvement. *Eur J Nutr* **51**, 173–179 (2012).

21. Vigotsky, A. D., Schoenfeld, B. J., Than, C. & Brown, J. M. Methods matter: The relationship between strength and hypertrophy depends on methods of measurement and analysis. *PeerJ* (2018) doi:10.7717/peerj.5071.

22. Biagi, E. *et al.* Through Ageing, and Beyond: Gut Microbiota and Inflammatory Status in Seniors and Centenarians. *PLoS ONE* **5**, e10667 (2010).

23. Ridaura, V. K. *et al.* Gut microbiota from twins discordant for obesity modulate metabolism in mice. *Science* **341**, 1241214 (2013).

24. Smith, P. *et al.* Regulation of Life Span by the Gut Microbiota in The Short-Lived African Turquoise Killifish. *bioRxiv* 120980 (2017) doi:10.1101/120980.

25. Buigues, C. *et al.* Effect of a Prebiotic Formulation on Frailty Syndrome: A Randomized, Double-Blind Clinical Trial. *International journal of molecular sciences* **17**, (2016).

26. Theou, O. *et al.* Can a Prebiotic Formulation Reduce Frailty Levels in Older People? *The Journal of frailty & aging* **8**, 48–52 (2019).

27. Walker, S. N. *et al.* Maintenance of activity and eating change after a clinical trial of tailored newsletters with older rural women. *Nurs Res* **59**, 311–321 (2010).

28. Cesari, M. *et al.* Added Value of Physical Performance Measures in Predicting Adverse Health-Related Events: Results from the Health, Aging, and Body Composition Study. *J Am Geriatr Soc* **57**, 251–259 (2009).

29. Harvey, N. C. *et al.* Measures of Physical Performance and Muscle Strength as Predictors of Fracture Risk Independent of FRAX, Falls, and aBMD: A Meta-Analysis of the Osteoporotic Fractures in Men (MrOS) Study. *Journal of Bone and Mineral Research* **33**, 2150–2157 (2018).

30. RStudio Team. RStudio: Integrated Development for R. *RStudio, Inc., Boston, MA* (2015).

31. Welch, A. A. Nutritional influences on age-related skeletal muscle loss. *Proceedings of the Nutrition Society* **73**, 16–33 (2014).

32. Deutz, N. E. P. *et al.* Protein intake and exercise for optimal muscle function with aging: Recommendations from the ESPEN Expert Group. *Clin Nutr. 2014* **33**, 929–936 (2014).

33. Biagi, E. *et al.* Through Ageing, and Beyond: Gut Microbiota and Inflammatory Status in Seniors and Centenarians. *PLoS One* **5**, e10667 (2010).

34. Ridaura, V. K. *et al.* Gut microbiota from twins discordant for obesity modulate metabolism in mice. *Science (1979)* **341**, 1241214 (2013).

35. E9 Expert Working Group. ICH Harmonised Tripartite Guideline. Statistical principles for clinical trials. International Conference on Harmonisation. *Stat Med* **18**, 1905–42 (1999).

36. Ni Lochlainn, M. *et al.* The PROMOTe study: targeting the gut microbiome with prebiotics to overcome age-related anabolic resistance: protocol for a double-blinded, randomised, placebo-controlled trial. *BMC Geriatr* **21**, 407 (2021).

37. Bowyer, R. C. E. *et al.* Use of dietary indices to control for diet in human gut microbiota studies. *Microbiome* **6**, 77 (2018).

38. Searle, S. D., Mitnitski, A., Gahbauer, E. A., Gill, T. M. & Rockwood, K. A standard procedure for creating a frailty index. *BMC Geriatr* **8**, 24 (2008).

39. Cox, N. J. *et al.* The composition of the gut microbiome differs among community dwelling older people with good and poor appetite. *J Cachexia Sarcopenia Muscle* **12**, 368–377 (2021).

40. Wilson, M.-M. G. M. G. *et al.* Appetite assessment: simple appetite questionnaire predicts weight loss in community-dwelling adults and nursing home residents. *Am J Clin Nutr* **82**, 1074–1081 (2005).

41. Ipaq.ki.se. *Guidelines for Data Processing and Analysis of the International Physical Activity Questionnaire (IPAQ)-Short Form*. www.ipaq.ki.se. (2004).

42. CRAIG, C. L. *et al.* International Physical Activity Questionnaire: 12-Country Reliability and Validity. *Med Sci Sports Exerc* **35**, 1381–1395 (2003).

43. Cruz-Jentoft, A. J. *et al.* Sarcopenia: revised European consensus on definition and diagnosis. *Age Ageing* **48**, 16–31 (2019).

44. Black, A. E. The sensitivity and specificity of the Goldberg cut-off for EI:BMR for identifying diet reports of poor validity. *Eur J Clin Nutr* **54**, 395–404 (2000).

45. Black, A. E. Critical evaluation of energy intake using the Goldberg cut-off for energy intake:basal metabolic rate. A practical guide to its calculation, use and limitations. *Int J Obes* **24**, 1119–1130 (2000).

46. Roza, A. M. & Shizgal, H. M. The Harris Benedict equation reevaluated: resting energy requirements and the body cell mass. *Am J Clin Nutr* **40**, 168–182 (1984).

47. Dietary reference values for food energy and nutrients for the United Kingdom. Report of the Panel on Dietary Reference Values of the Committee on Medical Aspects of Food Policy - PubMed. https://pubmed.ncbi.nlm.nih.gov/1961974/.

48. RStudio Team. RStudio: Integrated Development for R. *RStudio, Inc., Boston, MA* (2015).

49. Kahan, B. C., Jairath, V., Doré, C. J. & Morris, T. P. The risks and rewards of covariate adjustment in randomized trials: an assessment of 12 outcomes from 8 studies. *Trials* **15**, 139 (2014).

50. White, I. R., Horton, N. J., Carpenter, J., Statistics, r. i. m. a. s. & Pocock, S. J. Strategy for intention to treat analysis in randomised trials with missing outcome data. *BMJ* **342**, d40–d40 (2011).
