# Supplementary material for: Effect of gut microbiome modulation on muscle function and cognition: the PROMOTe randomised controlled trial
Source: Nat Commun. 2024 Feb 29;15:1859. doi: 10.1038/s41467-024-46116-y (PMC10904794; doi:10.1038/s41467-024-46116-y)
Supplement: Supplementary file 3 — Reporting Summary [file 41467_2024_46116_MOESM3_ESM.pdf]

nature portfolio

Corresponding author(s): Mary Ni Lochlainn

Last updated by author(s): Jan 5, 2024

Reporting Summary

Nature Portfolio wishes to improve the reproducibility of the work that we publish. This form provides structure for consistency and transparency in reporting. For further information on Nature Portfolio policies, see our Editorial Policies and the Editorial Policy Checklist.

Statistics

For all statistical analyses, confirm that the following items are present in the figure legend, table legend, main text, or Methods section.

n/a	Confirmed	
☐	☒	The exact sample size (*n*) for each experimental group/condition, given as a discrete number and unit of measurement
☒	☐	A statement on whether measurements were taken from distinct samples or whether the same sample was measured repeatedly
☐	☒	The statistical test(s) used AND whether they are one- or two-sided<br>*Only common tests should be described solely by name; describe more complex techniques in the Methods section.*
☐	☒	A description of all covariates tested
☐	☒	A description of any assumptions or corrections, such as tests of normality and adjustment for multiple comparisons
☐	☒	A full description of the statistical parameters including central tendency (e.g. means) or other basic estimates (e.g. regression coefficient) AND variation (e.g. standard deviation) or associated estimates of uncertainty (e.g. confidence intervals)
☐	☒	For null hypothesis testing, the test statistic (e.g. *F*, *t*, *r*) with confidence intervals, effect sizes, degrees of freedom and *P* value noted<br>*Give P values as exact values whenever suitable.*
☒	☐	For Bayesian analysis, information on the choice of priors and Markov chain Monte Carlo settings
☒	☐	For hierarchical and complex designs, identification of the appropriate level for tests and full reporting of outcomes
☐	☒	Estimates of effect sizes (e.g. Cohen's *d*, Pearson's *r*), indicating how they were calculated

Our web collection on statistics for biologists contains articles on many of the points above.

Software and code

Policy information about availability of computer code

Data collection

A web based electronic data capture (EDC) system was designed, using the InferMed Macro 4 system. The EDC was created in collaboration with the trial analysts and the Principal Investigator and maintained by the King's Clinical Trials Unit for the duration of the project. It was hosted on a dedicated server within King's College London.

Database access was strictly restricted through user-specific passwords to the authorised research team members. Participant initials and date of birth were entered on the EDC, NHS number, email addresses, participant names and addresses, and full postcodes were not entered into the EDC. No data was entered onto the EDC system unless a participant has signed a consent form to participate in the trial. Source data was entered by recruiting site staff, typically within 5 days of data collection by authorised staff onto the EDC. A full audit trail of data entry and any subsequent changes to entered data was automatically date and time stamped, alongside information about the user making the entry/changes within the system. The study team undertook appropriate reviews of the entered data, for the purpose of data cleaning.
At the end of the trial, the PI reviewed all the data for each participant to verify that all the data are complete and correct. At this point, all data was formally locked for analysis.

All questionnaire and video teleconferencing visit data were inputted to the EDC database.
For dietary data collection, the online myfood24 dietary reporting software was used.
Cognitive testing was carried out using the online CANTAB cognitive battery, and results downloaded from CANTAB website in .csv format.

Stool and blood samples were collected by the participants themselves using the sample collection kits provided to them in the postal pack. All sample kits provided, and participant data were pseudo-anonymised with a unique identifier, the samples barcoded to link with participants unique ID. The principal investigator ensured that all data collected in the study, was recorded in a timely manner

according to any instructions provided and the subject numbering process commenced at the point of informed consent. All sample collection kits provided to the participants contain detailed instruction on storage conditions to ensure the integrity and viability of samples. Participants were asked to record the date and time of collection for each sample they collected. The samples sent back to the laboratory were logged in using the unique barcode and/or unique ID. A complete chain-of-custody was maintained for all samples throughout, from point of acquisition, storage and all uses, including disposal where relevant.

DNA extracted from faecal samples was sent to Clinical Microbiomics for microbiome analysis. A material transfer agreement was in place between the investigators, the sponsors, the host laboratory at King's College London, and the third party laboratory.

Data analysis

Analysis was performed using Stata SE version 15.1, other than gut microbiome analysis, which was performed using R version 4.2.1.
Gut microbiome analysis utilised R packages vegan (available from: https://cran.r-project.org/web/packages/vegan/index.html) and mets (available from https://cran.r-project.org/web/packages/mets/index.html).

The tool CheckM was used to process each metagenomic species (available from ecogenomics.github.io/CheckM/).
The algorithm AdapterRemoval (v 2.3.2) was used to trim reads to remove adapters and based with a Phred score below 20 (available from adapterremoval.readthedocs.io/).
The tool EggNOG-mapper (v 2.0.1) was used for functional annotation of the sequences (available from eggnog-mapper.embl.de)
Testing for differences in microbiome taxon abundances was performed with a linear regression framework with a compositional bias correction based on LinDA (See: Zhou et al. 2022 (PMID:35421994)).
Pairwise comparisons between study groups were performed by contrasting the estimated marginal means for each study group with the R package "emmeans" (avaialble from https://cran.r-project.org/web/packages/emmeans/index.html).
Broad-sense heritability was determined with ACE model (from R package mets), (available from https://cran.r-project.org/web/packages/mets/index.html).

For manuscripts utilizing custom algorithms or software that are central to the research but not yet described in published literature, software must be made available to editors and reviewers. We strongly encourage code deposition in a community repository (e.g. GitHub). See the Nature Portfolio guidelines for submitting code & software for further information.

Data

Policy information about availability of data

All manuscripts must include a data availability statement. This statement should provide the following information, where applicable:

- Accession codes, unique identifiers, or web links for publicly available datasets
- A description of any restrictions on data availability
- For clinical datasets or third party data, please ensure that the statement adheres to our policy

Data are available on request from TwinsUK, please see https://twinsuk.ac.uk/resources-for-researchers/access-our-data/. for details.
Raw sequencing data is available in the UK Data Service (ReShare) depository. Access link: https://dx.doi.org/10.5255/UKDA-SN-856726

Microbiome Anaylysis Methods:
For DNA sequencing, the fragmented DNA was used for library construction using NEBNext Ultra II Library Prep Kit for Illumina (New England Biolabs).
We used a reference gene catalogue with 14 355 839 genes created from 21 598 human gut specimens (including 481 from infants, 9 428 publicly available metagenomes compiled from 43 countries) [Pasolli et al. 2019, PMID: 30661755].
To taxonomically annotate an MGS, we blasted its genes against NCBI RefSeq prokaryotic genomes (2022-01-19) and nt (2021-08-03) databases
The human reference genome Bowtie2 (v. 2.4.2) was used in filtering of the raw FASTQ files.
The gene catalogue BWA mem (v 0.7.17) was used for mapping high-quality non host reads.
The Kyoto Encyclopaedia of Genes and Genomes (KEGG) orthology (KO) database (v 78.2) was utilised for functional annotation and profiling, as well as the EggNOG (v. 5.0) orthologous groups database. EggNOG (v 3.0) was used to define gut brain modules, and identify seed orthologs for non-supervised orthologous groups. The TIGRFAMs database of protein families was also reviewed in analysis of gut brain modules.

Research involving human participants, their data, or biological material

Policy information about studies with human participants or human data. See also policy information about sex, gender (identity/presentation), and sexual orientation and race, ethnicity and racism.

Reporting on sex and gender

The study recruited male and female adult participants as twin pairs. All twin pairs were same-sex. Most participants in the study were female (56/72; 78%). No other sex-specific analyses were carried out due to small sample sizes. No gender information was collected. Sex was self-reported.

Reporting on race, ethnicity, or other socially relevant groupings

Demographic information and baseline characteristics were collected in the baseline questionnaire. Demographic information is also collected when participants first register as volunteers within the TwinsUK longitudinal cohort. Standard demographic parameters include age, sex, and race/ethnicity (collected in accordance to prevailing regulations). As this information is already collected as part of TwinsUK, this was not re-collected as part of the PROMOTe study.

Population characteristics

Participants were eligible for inclusion if they were aged 60 years or older and had previously reported low dietary protein intake (<1g/kg body weight/day) according to the European Society for Clinical Nutrition and Metabolism (ESPEN) guidance for older adults 7. This was chosen over the Recommended Nutrient Intake (RNI) for protein in the UK (0.8g/kg/day) due to anabolic resistance of skeletal muscle associated with ageing. Intake of protein to determine entry to the study was measured a priori within the TwinsUK cohort. In addition, participants had to have access to a computer or tablet device, in order to be able to complete the remote visits via video teleconference. The exclusion criteria included severe food allergy, current or recent (preceding 3 months) use of antibiotics, protein supplements, prebiotics or probiotics, chronic kidney disease stage 3 or higher, weight loss of ≥5% of body weight in the preceding 12 months, any significant injury or surgery

	which currently affects physical functioning, and current involvement in other interventional studies. These criteria were selected to avoid contraindications to the interventions, to avoid contamination of data collected for example with recent use of a protein supplement, and to maximise reliability of the gut microbiota samples collected, for example by excluding recent antibiotic use and significant gastrointestinal disease.<br><br>The TwinsUK cohort is majority female for historical reasons, and therefore it was unsurprising to have majority female participants. The demographic characteristics of those who were ineligible or declined to take part, was compared to those who took part, using existing TwinsUK longitudinal data. Participants were younger and there were more males than the wider TwinsUK cohort. The arms were well matched at baseline, as expected in a twin study, however there was a difference in the two groups for SNAQ appetite score, thus this was included in the subsequent analyses as a potential confounder.<br>This study aimed to look at over 60s, and therefore is not generalisable to younger age groups.
Recruitment	Participants were selected from the existing TwinsUK cohort based on age, and protein intake data - due to low protein intake and age >60 years being inclusion criteria for the study. Once identified as potentially eligible, participants were contacted by phone and screened for further inclusion/exclusion criteria and whether they were interested in taking part. Information sheets were provided to those interested and eligible, and they then went on to the consenting process.<br><br>In comparison to those who were ineligible to chose not to take part, study participants were more likely to be younger (mean age 75(5) versus 73(5), p=0.006), and more likely to be male (9% of declined/ineligible vs 22% of participants, p<0.001). It is possible that the slightly younger profile in influenced by the reduced digital literacy of the older twins who declined to take part, and future studies should consider hybrid approaches to remotely delivered trials, to ensure equitable access to research for those who are not digitally literate, in keeping with NIHR INCLUDE guidance.
Ethics oversight	The PROMOTe Study was approved by NHS North of Scotland Research Ethics Service (REC reference 21/NS/0045), IRAS ID 257415. This study was carried out under TwinsUK BioBank ethics, approved by North West – Liverpool Central Research Ethics Committee (REC reference 19/NW/0187), IRAS ID 258513. This approval supersedes earlier approvals granted to TwinsUK by the St Thomas' Hospital Research Ethics Committee, later London – Westminster Research Ethics Committee (REC reference EC04/015), which have now been subsumed within the TwinsUK BioBank.<br>Written informed consent was obtained from all participants. All research therefore carried out in accordance with the ethical standards laid down in the 1964 Declaration of Helsinki and its later amendments.

Note that full information on the approval of the study protocol must also be provided in the manuscript.

Field-specific reporting

Please select the one below that is the best fit for your research. If you are not sure, read the appropriate sections before making your selection.

☒ Life sciences ☐ Behavioural & social sciences ☐ Ecological, evolutionary & environmental sciences

For a reference copy of the document with all sections, see nature.com/documents/nr-reporting-summary-flat.pdf

Life sciences study design

All studies must disclose on these points even when the disclosure is negative.

Sample size	From existing data within TwinsUK cohort, we observed that chair-rise time was approximately log normal, with log10(chair rise time) having a SD of 0.126. We considered a relative reduction in chair rise time of 20% to be both plausible and clinically important. We based this on previous studies using chair rise time 32–34, however it was noted that no study had investigated this in the context of a gut microbiome-focused intervention. Based on these figures, we computed that we would need complete data on 28 participants per group (56 in total) for 80% power. Allowing for 20% dropouts, we needed 67.2 participants recruited , and rounded this up to n=70 (35 per group) for ease. Using twins increases study power due to close matching at baseline, with reduced genetic and/or environmental variability, and therefore reduced confounding.
Data exclusions	No data were excluded.
Replication	Replication of the findings of this clinical trial could not be reproduced as this would entail another trial and further research ethical approval. We hope to conduct a larger trial in future to confirm these findings.
Randomization	Randomisation and arm-allocation was done remotely by the Kings Clinical Trials Unit, who issued the corresponding number for the correct sachets to be sent to each participant by the research team. Randomisation was done as twin pairs, each pair as a fixed block of two – one twin in each pair randomly allocated to each arm. All participants and researchers were blinded until data analysis was complete.
Blinding	All participants and researchers were blinded until data analysis was complete.

Reporting for specific materials, systems and methods

We require information from authors about some types of materials, experimental systems and methods used in many studies. Here, indicate whether each material, system or method listed is relevant to your study. If you are not sure if a list item applies to your research, read the appropriate section before selecting a response.

Materials & experimental systems

n/a	Involved in the study
☒	☐ Antibodies
☒	☐ Eukaryotic cell lines
☒	☐ Palaeontology and archaeology
☒	☐ Animals and other organisms
☐	☒ Clinical data
☒	☐ Dual use research of concern
☒	☐ Plants

Methods

n/a	Involved in the study
☒	☐ ChIP-seq
☒	☐ Flow cytometry
☒	☐ MRI-based neuroimaging

Clinical data

Policy information about clinical studies

All manuscripts should comply with the ICMJEguidelines for publication of clinical research and a completedCONSORT checklist must be included with all submissions.

Clinical trial registration: ClinicalTrials.gov registration: NCT04309292.

Study protocol: Included in supplementary material.

Data collection: Recruitment opened in May 2021. The date of the first visit for the first participant was 17/05/2021, and the date of the second (final) visit of the last participant was 20/12/2021.
Data was collected remotely - via online questionnaires, online food diaries, online cognitive testing, video teleconferencing visits, and remote biological sample collection (via post).

Outcomes: Outcomes were collected at the study visits via video teleconferencing, via remote completion of questionnaires, three-day food diary to measure protein and other dietary intake to account for any differences in intake that would lead to confounding, and online cognitive test battery, and via postal receipt of biological samples from participants.
The primary outcome was change in chair rise time (time to do 5 chair rises without using arms), measured at baseline and study end. Chair rise time is a component of the short physical performance battery (SPPB), and is associated with quality of life, physical function, frailty, multimorbidity and indeed, cognitive function. It is the recommended measure of the strength in the European Working Group of Sarcopenia of Older Persons 2 guidance.
Secondary outcomes included cognitive battery factor score, SPPB score (includes chair rise time and gait speed), grip strength, gait speed, self-reported physical activity levels using the International Physical Activity Questionnaire (IPAQ) and Simplified Nutritional Assessment Questionnaire (SNAQ) appetite score.

Plants

Seed stocks: n/a

Novel plant genotypes: n/a

Authentication: n/a
